# Determination of Multidirectional Pathways for Ligand Release from the Receptor: A New Approach Based on Differential Evolution

**DOI:** 10.1021/acs.jctc.1c01158

**Published:** 2022-05-05

**Authors:** Hoang Linh Nguyen, Nguyen Quoc Thai, Mai Suan Li

**Affiliations:** † Life Science Lab, Institute for Computational Science and Technology, QuangTrung Software City, Tan Chanh Hiep Ward, District 12, Ho Chi Minh City 729110, Vietnam; ‡ Ho Chi Minh City University of Technology (HCMUT), Ho Chi Minh City 740500, Vietnam; § Vietnam National University, Ho Chi Minh City 71300, Vietnam; ∥ Dong Thap University, 783 Pham Huu Lau Street, Ward 6, Cao Lanh City, Dong Thap 81100, Vietnam; ⊥ Institute of Physics, Polish Academy of Sciences, Al. Lotnikow 32/46, Warsaw 02-668, Poland

## Abstract

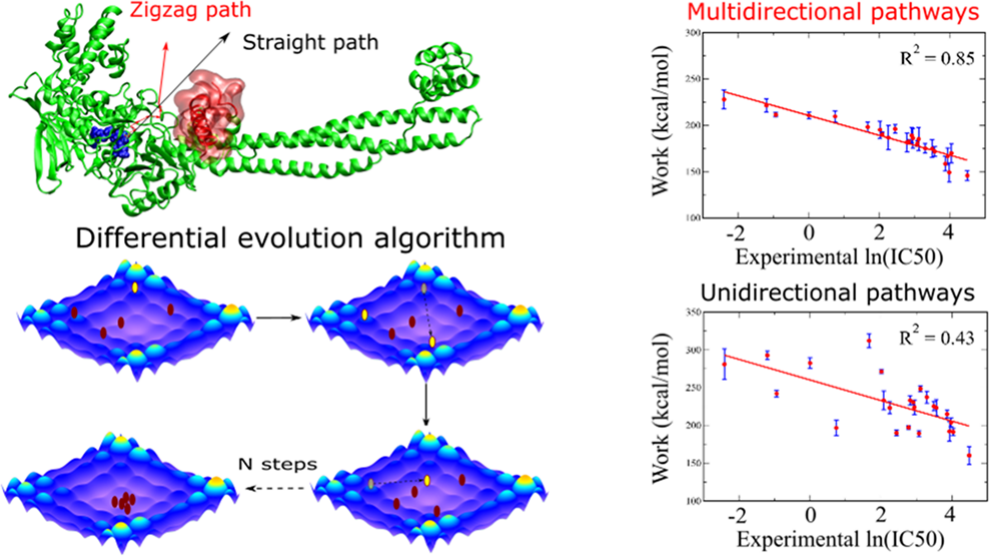

Steered molecular dynamics (SMD) simulation is a powerful method in computer-aided drug design as it can be used to access the relative binding affinity with high precision but with low computational cost. The success of SMD depends on the choice of the direction along which the ligand is pulled from the receptor-binding site. In most simulations, the unidirectional pathway was used, but in some cases, this choice resulted in the ligand colliding with the complex surface of the exit tunnel. To overcome this difficulty, several variants of SMD with multidirectional pulling have been proposed, but they are not completely devoid of disadvantages. Here, we have proposed to determine the direction of pulling with a simple scoring function that minimizes the receptor–ligand interaction, and an optimization algorithm called differential evolution is used for energy minimization. The effectiveness of our protocol was demonstrated by finding expulsion pathways of Huperzine A and camphor from the binding site of Torpedo California acetylcholinesterase and P450cam proteins, respectively, and comparing them with the previous results obtained using memetic sampling and random acceleration molecular dynamics. In addition, by applying this protocol to a set of ligands bound with LSD1 (lysine specific demethylase 1), we obtained a much higher correlation between the work of pulling force and experimental data on the inhibition constant IC50 compared to that obtained using the unidirectional approach based on minimal steric hindrance.

## Introduction

Determining the binding affinity of a ligand to a receptor is an important problem in drug development. Various computational methods are used to solve this problem, starting with molecular docking,^1^ which is fast but not accurate enough due to insufficient sampling. However, due to its high computation speed, it is widely used for the initial screening of potential compounds from large databases. More precise methods are based on molecular dynamics (MD) modeling, including free energy perturbation, thermodynamics integration (TI), molecular mechanics Poisson–Boltzmann surface area (MM-PBSA), molecular mechanics generalized Born surface area (MM-GBSA) methods,^2^ and so forth.

Beside binding affinity, binding and unbinding kinetics and ligand pathways provide important information about drug efficacy. Many methods have been developed to investigate the association and dissociation of the ligand–protein complex.^3−16^ Using concurrent adaptive sampling, which is a weighted ensemble approach, Ahn et al. ranked ligand-binding kinetics for target β-cyclodextrin.^17^ Wolf et al. studied the ligand dissociation dynamics using targeted molecular dynamics (TMD) simulations with correction for dissipation.^18,19^ Metadynamics is another approach to access kinetic profiles,^20−22^ while You and Chang combined several methods to study the dissociation process.^23^ In particular, funnel metadynamics^24^ can produce not only binding free energy but also unbinding pathways. Furthermore, machine learning emerges as a promising approach to enhance sampling related to ligand dissociation.^25^

The steered molecular dynamics (SMD)^26−29^ was first implemented to probe the molecular mechanisms of biomolecular processes studied using single-molecule spectroscopy techniques such as atomic force microscopy (AFM) and laser optical tweezers.^30,31^ Later, SMD was employed to investigate the protein–ligand interaction by various groups.^32−38^ Today, various modifications such as hybrid SMD,^39^ ensemble-based SMD,^40^ DelPhiForce SMD,^41^ and so forth have been developed. The influence of the pulling velocity and the number of simulation trajectories on the prediction of protein–ligand affinity was examined.^42^

Recently, it has been shown that SMD is as accurate as MM/PBSA^43^ but fast enough to deal with a large number of ligands.^34−36,39,43,44^ It should be noted that if SMD is combined with Jarzynski equality^45^ to calculate the absolute equilibrium free energy, then SMD takes more time than MM/PBSA. However, when SMD is used at a high pulling speed to obtain the relative binding affinity, by comparing the pulling work, SMD is observed to be faster.^34−36,39,43,44^

SMD results depend on the pulling direction, which can be either unidirectional or multidirectional. A unidirectional pulling path can be obtained using different methods such as CAVER,^46^ MOLE,^47^ and MSH (minimal steric hindrance),^48^ which yield the agreement between the simulation and experiment for many systems^32,34,49−51^ (note that MSH is based on SMD simulations, while CAVER and MOLE identify paths using a static structure). However, as shown below, even MSH will lead to a low correlation between the pulling work, which can be used to distinguish binders from nonbinders,^48^ and the experimental IC50 values of 24 compounds bound to the anti-cancer therapy target LSD1 (lysine specific demethylase 1).^52^ One of the possible reasons for SMD failure in this case is that LSD1 has a narrow exit channel that is not suitable for pulling the ligand along a straight pathway, which prompts us to try multidirectional pulling.

There are several methods to find the best zigzag direction. In the random acceleration MD (RAMD) method,^53^ a force of random direction is applied on the ligand, which may or may not be updated according to the ligand displacement in a certain period of time. This process continues until the ligand is released from the receptor. However, due to the random choice of the force orientation, the success rate in finding egress routes may be low compared to that in other methods.^54^ Here, the success rate is defined as the percentage of trajectories leading to a ligand exit. A recent development of RAMD is τ_RAMD_,^55,56^ which only requires the magnitude of the random force as a parameter for ranking the residence times of diverse sets of compounds. Another method for finding the ligand binding pathway is the string method, which interpolates intermediate states between bound and unbound states.^57,58^ Combining RAMD, SMD, and umbrella sampling, it has been shown that very long simulations are needed in order to obtain an accurate ranking of the distinct access and egress routes and the free energy profiles.^59,60^

Rydzewski et al. proposed two memetic algorithms called memory random acceleration (MERA) and the immune algorithm (IA) to find the optimal ligand egress pathway.^54,61^ In MERA, each position of the ligand leaves a trace for the next attempt. The ligand is pulled by a random force, which depends on the concentration of the traces. This algorithm is equivalent to the ant colony optimization algorithm. The MERA method requires an initial trace distribution before performing MERA and MD simulation. This distribution can be obtained by using RAMD or locally enhanced sampling.^61^ In the IA, the objective function, which is the interaction energy between the ligand and the protein, is obtained using the Hammett linear free energy function.^54^ This algorithm searches for a path that has the minimum interaction energy at each pulling step, and the pulling direction coincides with this path. MERA and the IA have a higher success rate of pulling out the ligand than RAMD.^54^ The IA has been successfully used to find egress pathways of camphor from cytochrome P450cam.^62^ Rydzewski and Valsson^64^ recently proposed a method for finding pathways for ligand unbinding using convex optimization of a function that describes the protein–ligand interaction.

To solve this problem, we can find a multidirectional pathway using one of the methods mentioned above^53,54,61,63,64^ because popular RAMD is built into major software packages such as NAMD,^65^ AMBER,^66,67^ and GROMACS,^56^ while the code for the recent work of Rydzewski et al.^64^ (maze) is available on PLUMED.^68^ However, since SMD is a valuable tool in predicting the relative binding of a ligand, it would be interesting to use this method to find multidirectional pathways for ligand egress from the protein active site. Such a SMD-based approach has been developed by several groups.^69,70^ Yang et al.^69^ combined on-the-fly SMD with a multi-population genetic algorithm to find minimal energy paths. Gu et al.^70^ built the objective function based on the criterion that the best path provides the least rupture force. Here, the multi-population genetic algorithm was also used to solve the optimization problem. In this approach, the initial direction is chosen manually, and its population may be small, affecting the efficiency of the protocol.

Since zigzag dissociation channels have not been studied in detail,^69,70^ it is unclear if the on-the-fly SMD approach can reveal all pathways in complex systems such as the P450cam-camphor complex. In addition, the code related to the adaptive SMD^70,71^ is not available online and is not easy to implement, prompting us to develop our own protocol based on differential evolution (DE)^71^ to determine a multidirectional pathway.

Our tool interacts with the GROMACS package through a bash shell script. We showed that our in-house program provides pathways that have been observed using previous methods^53,62,72^ for the dissociation of Huperzine A (HupA) from Torpedo californica acetylcholinesterase (TcAChE) and camphor from P450cam. This indicates the reliability of our protocol in detecting zigzag pathways for ligand exit from the receptor-binding site. Moreover, our protocol significantly improved the correlation between non-equilibrium work and experimental IC50 data for LSD1, suggesting that it can be applied to study the binding affinity of a complex with a narrow channel.

## Materials and Methods

### Set of 24 Ligands Bound to LSD1

**Figure 1:**
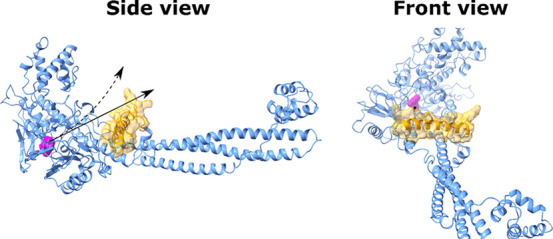
Schematic for the initial complex structure of LSD1 and the ligand (magenta). The pulling direction is shown as a black arrow in the side view and a black dot in the front view, which represents the direction points to the reader. The obstacle regions are show in yellow with a transparent surface. The dotted black arrow suggests a pathway that ligands can avoid clash with red regions.

We will study the binding affinity of 24 ligands to LSD1, the IC50 of which was experimentally measured (Table S1 in the Supporting Information). Note that the IC50 is not directly related to binding affinity but can be used to measure it since the IC50 is proportional to the dissociation constant *K*_*i*_ through the Cheng–Prusoff equation. The initial structure of LSD1 was obtained from the protein data bank (PDB) with PDB code 2UXN(73) (Figure 1). The structures of 24 ligands were retrieved from PubChem^74^ and ChemSpider^75^ (Table S1).

### Structures of P450cam-Camphor and TcAChE–HupA Complexes

To test our new protocol, we studied two cases: HupA binds to Acetylcholinesterase (AChE) and camphor binds to P450cam. In the first case, the structure of Torpedo California AChE (TcAChE) was obtained from the protein data bank (PDB) with PDB id 1AE5,^76^ while for the heme–camphor complex, we used the PDB structure with id 2CPP.^77^ The parameters for heme were collected from Shahrokh et al.^78^

### Molecular Docking

AutoDock Tools 1.5.4^79^ was used to prepare the input for docking simulation with the PDBQT format. The docking simulation is carried out using AutoDock Vina 1.5.4,^80^ where the ligand structure was flexible. The docking simulation box was centered at the binding site, and its grid size and grid center for all targets are shown in Table S2. To access good quality of the docking, the completeness parameter for global search was set to 600. The docking structure obtained in the lowest binding energy mode of the receptor–ligand complex was used as the initial configuration for SMD simulation.

### MD Simulations

MD simulations were carried out using the GROMACS 2018 package.^81^ The complex structure was solvated using the TIP3P water model^82^ in a rectangular box with a minimum distance of 2 nm between the protein and the box edges. Counter ions were added to neutralize the charge of the system. We used the AMBER99SB-ILDN^83^ force field to parameterize the protein.

The parameters of the ligand that binds to LSD1 were computed using Antechamber^84^ and Acpype,^85^ which are based on the general AMBER force field.^86^ Atomic point charges were determined using AM1-BCC.^87^ The system was relaxed using the steepest descent algorithm^88^ and was then equilibrated for 500 ps in an *NVT* ensemble at 300 K reserved using the *v*-rescale algorithm.^89^ It was then held at 1 bar using the Parrinello-Rahman algorithm for 5 ns in an *NPT* ensemble,^90^ and to avoid an abrupt change in the structure, the heavy atoms of the protein and ligand were restrained by a harmonic potential with a spring constant of 1000 kJ/mol/nm.^2^ Then, to achieve a properly equilibrated structure of the ligand, an additional 100 ns *NPT* simulation was performed without restriction. The cutoff for nonbonded interactions was 1.0 nm.

### SMD Simulation

In SMD, an external force is applied to a dummy atom that is linked to the ligand atom closest to the CoM of the ligand using a spring with a stiffness *k*. Then, the force experienced by the ligand is *F* = *k*(Δ*x* – *vt*), where *v* is the pulling speed and Δ*x* is the pulled atom displacement from the initial position. We chose *k* = 600 kJ/mol·nm^2^, which is the typical value used in the AFM experiment.^91^

From the force–time/position profile, we collect the rupture force *F*_max_, which is necessary for the dissociation of the protein–ligand complex and can be used to rank binding affinity. The pulling work *W*_pull_, which is another metric for comparing binding affinity, was calculated using the following formula
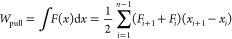
1where *F*_*i*_ and *x*_*i*_ are the force and displacement at step *i*, respectively, and *n* is the number of simulation steps. Since the work^48^ and rupture force^72,92^ depend on the pulling speed but the correlation between the simulation data, obtained in a highly non-equilibrium regime, and the experimental data does not depend on it,^48^ we used *v* = 5 nm/ns. For each protein–ligand complex, we performed 20 independent SMD trajectories in both unidirectional and multidirectional simulations, and *W*_pull_ was averaged over all trajectories. The number of trajectories was chosen so that the error bar, presented as the standard deviation, was less than or equal to 10% of the mean.

### Choice of the Pulling Pathway

We performed SMD simulations with unidirectional and multidirectional pulling pathways. In the unidirectional case, the ligand was pulled in the direction determined using the MSH algorithm.^48^ Such a pathway is shown in Figure 1 for LSD1 (black arrow). For each receptor–ligand complex, 20 SMD trajectories of 2 ns each were conducted.

The multidirectional pathway was found using our new protocol, which is described in the Results and Discussion section. In this case, the pulling direction was changed every 20 ps, and the rationale for this choice is also shown in the Results and Discussion section.

### Pearson Correlation Coefficient

The Pearson correlation coefficient between the pulling work *W* and experimental IC50 values was calculated as follows
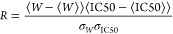
2where *X* and σ_*X*_ represent the average and standard deviation of quantity *X*, respectively.

## Results and Discussion

### Unidirectional SMD Simulation Provides a Poor Correlation with Experimental IC50 of LSD1–Ligand Complexes

Our docking simulation showed that all 24 ligands have the same binding site in LSD1 (Figure S1). Consequently, the pulling directions obtained using the MSH method are almost the same since the exit tunnel is narrow.

**Figure 2:**
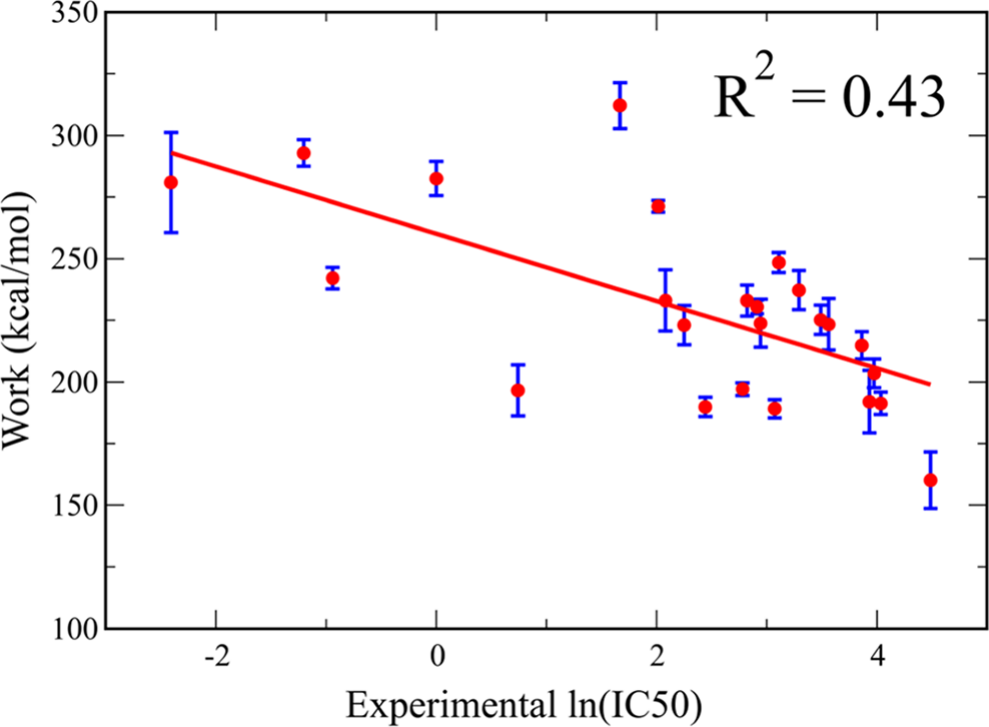
Pulling work obtained from straight pulling direction SMD simulations as a function of experimental ln(IC50) with IC50 measured in moles per liter. The red line represents the fitting function of data. *R*^2^ indicates the correlation coefficient. The results were averaged over 20 SMD trajectories, and error bars represent standard deviations.

Previous studies^49,93^ have shown that pulling work *W*_pull_ obtained from SMD simulations correlates better with experimental IC50 values than the rupture force (maximum force in the force–extension/time profile). This is due to the fact that work is defined for the whole process, while the maximum force is achieved in one state. Therefore, in this study, we calculate work profiles for a set of 24 ligands bound with LSD1 (Figure S2), and *W*_pull_ is defined as work at the last point. As mentioned above, IC50 is not directly related to the binding free energy Δ*G*, but it is reasonable to assume that Δ*G* ∼ ln(IC50). Thus, we showed the correlation between *W*_pull_ and ln(IC50) (Figure 2) and obtained a moderate correlation level^94^ with the coefficient *R*^2^ = 0.43. To understand why the correlation between the SMD simulation and experiment is not high, we examined force–time profiles. For trajectories 2, 5, 9, and 10 of ligand 1, a second peak occurs after the major maximum (Figure S3), indicating a possible collision between the ligand and the receptor during its egress. A similar situation also occurred with other ligands (data not shown), which implies that a straight direction obtained by using the MSH protocol does not work well for the narrow exit tunnel, and this may result in a low correlation between the simulation and experiment. Furthermore, since MSH has been shown to have better performance^48^ than other similar methods,^46,47^ the existing unidirectional approaches cannot improve the correlation with the experiment predicted using MSH (*R*^2^ = 0.43).

The pulling directions obtained using the MSH algorithm are vectors that point from regions E308–A309 of chain A to regions G314–R316, A809–V811 of chain A and are finally directed to a location that is close to regions R308–G313 of chain B and K371–F396 of chain A (Figure S4).

Although the external force does not direct the ligand to pass through the R308–G313 and K371–F396 regions of LSD1, the side chains of these regions can interfere with the ligand movement due to their proximity to the pulling path. This can be seen by the strong interaction between the ligand and these areas (Figure S3), the minima of which are close to the second peak of the force experienced by the ligand [see Figure S3 where the distance between the center of mass (CoM) of the ligand and the CoM of regions R308–G313 and K371–F396 of LSD1 is shown]. The average of minimum distance of all ligands is about 13.9 ± 1.0 Å, which indicates that the ligands approach closer to these regions during SMD simulation.

Since the collision problem in the R308–G313 and K371–F396 regions arises when a unidirectional pathway is used, we expect this problem to be avoided if the ligand moves along the orange path (Figure 1). However, in unidirectional pulling, if these regions can be avoided, the ligand may interfere with other protein residues on the surface of the binding pocket. Therefore, a different approach is required to find a pathway that retains the ligand both from the binding pocket surface and from the R308–G313 and K371–F396 regions.

### Adaptively Changing Direction in SMD Simulations

**Figure 3:**
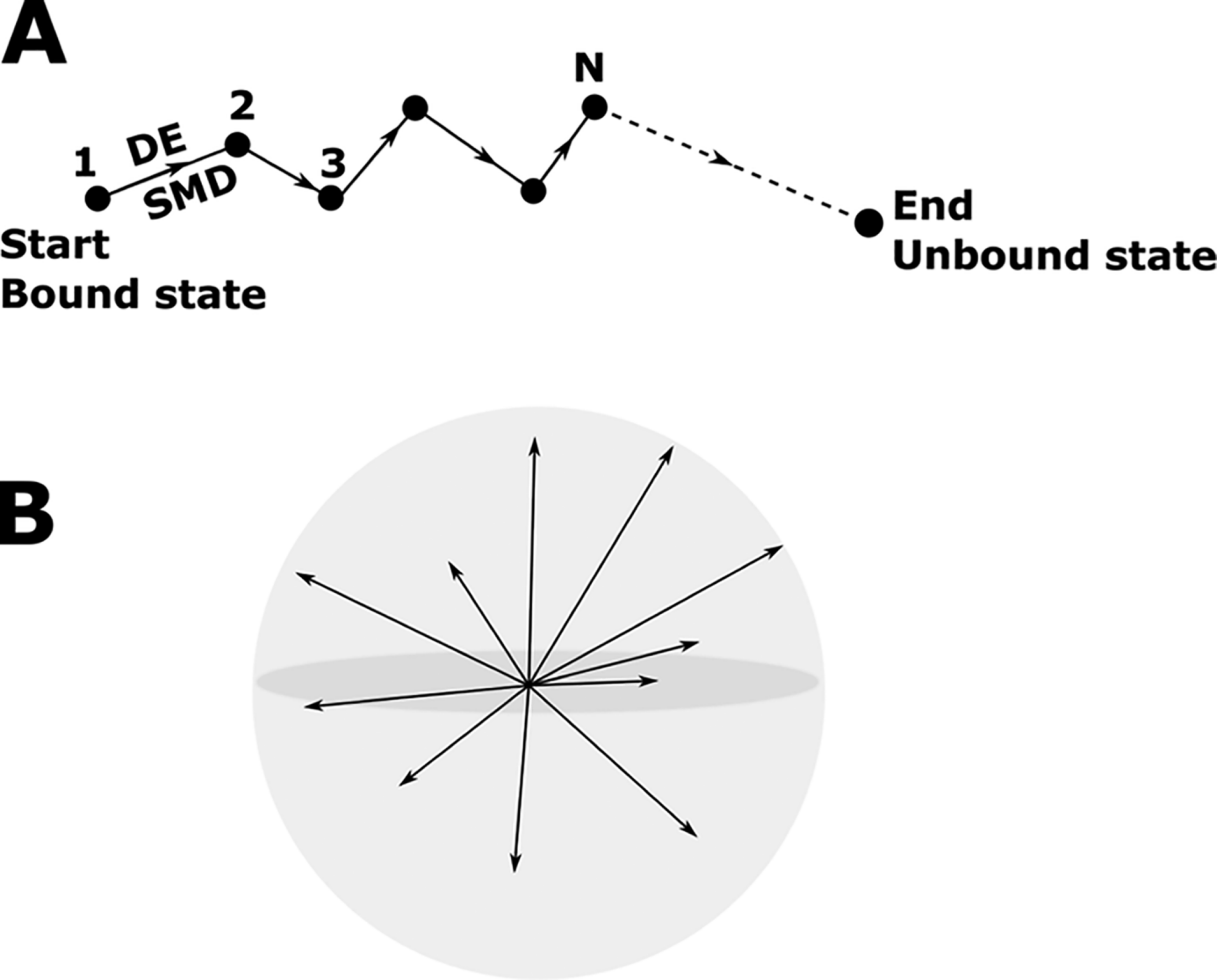
(A) Schematic description of our method, which involves *N* intervals to transition from a bound state in a binding site to an unbound state. One interval corresponds to the time interval τ = 20 ps of SMD simulation at a pulling speed of 5 nm/ns. For each step, we first applied DE to identify the short straight path, and once that path was found, SMD was performed to pull the ligand from point *i* to point *i* + 1. This procedure was repeated until the ligand reached the unbound state. (B) Representation of 3000 vectors randomly generated in DE. The maximum vector length is 7 Å.

The basic idea of our method is that to get the pulling direction on the fly in SMD simulations, we split the simulations into *N* short intervals (Figure 3A). Below, we show that the time interval τ = 20 ps is optimal in our method, when SMD was carried out at a pulling speed of 5 nm/ns. For each interval, the so-called DE^71^ was used to identify the optimal unidirectional path. Once such a path was found, we used SMD to transfer the ligand from point *i* to point *i* + 1. This process is repeated *N* times until the ligand reaches an unbound state in solution (Figure 3A). *N* is equal to the simulation time τ_sim_ divided by the time interval τ = 20 ps (*N* = τ_sim_/τ) and depends on the system (Table S3) in the range from 50 (LSD1) to 150 (TcAChE). To find the exit paths, we will execute 20 trajectories for LSD1 and TcAChE and 60 trajectories for P450cam (Table S3). The duration of each trajectory is usually shorter than the cutoff time τ_sim_, resulting in practical steps less than the *N* indicated in Table S3 (see below).

To find an optimal path by using DE, we define the scoring *E*_score_ as follows
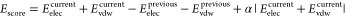
3where *E*_elec_ and *E*_vdw_ are the electrostatic and van der Waals interaction energies between the ligand and receptor, respectively. The upper “current” refers to the probe configuration that is generated from the random translation of the ligand, while the “previous” refers to the configuration obtained at the end of a short step in the SMD simulation. At each short step, we calculate the minimum of *E*_score_, which is the local energy.

Why do we need an α|*E*_elec_^current^ + *E*_vdw_^current^| term if we can assume that *E*_score_ = *E*_elec_^current^ + *E*_vdw_^current^ – *E*_elec_^previous^ – *E*_vdw_^previous^? Imagine that we get into a global minimum or some state with very low energy, then the searching procedure would stop, but adding this term helps avoid the trap. Term α|*E*_elec_^current^ + *E*_vdw_^current^| can also prevent the ligand and receptor overlap.

To settle the coefficient α, we carried out, for example, 20 SMD trajectories for the TcAChE–HupA complex with α = 0, 1, 2, 3, and 4. The numbers of the obtained paths are 10, 4, 6, 4, and 2 for α = 0, 1, 2, 3, and 4, respectively, which shows that α values of 0 and 2 give more paths than other values of α. However, the camphor ligand gets stuck at several positions along the SMD trajectory for the P450cam–camphor complex with α = 0. Thus, we chose α = 2 to balance the ability to capture rare paths and prevent ligand stuck. Force field parameters of atoms are the same as those in conventional MD simulations.

In our method, the direction in each step is determined corresponding to the minimum *E*_score_. From this point of view, our method differs from RAMD,^53^ where the direction is chosen at random and changes if the ligand encounters the cavity surface of the protein. In addition, the success rate of the RAMD depends on the constant force and threshold velocity, which are not easy to select,^61^ but this disadvantage was addressed in the τ_RAMD_ protocol^55^ by providing a rather straightforward procedure for determining the parameters of the random force for protein–ligand dissociation.

Our score function (eq 3) is also different from the other studies that use optimization algorithms. For instance, Gu et al. used information entropy to model a multi-objective problem,^70^ and the initial pulling direction is not selected by these algorithms but manually to make sure that the ligand is pulled toward the solvent. In our algorithm, the objective function is simple to reduce the computational effort, and the program can automatically determine the initial direction.

**Figure 4:**
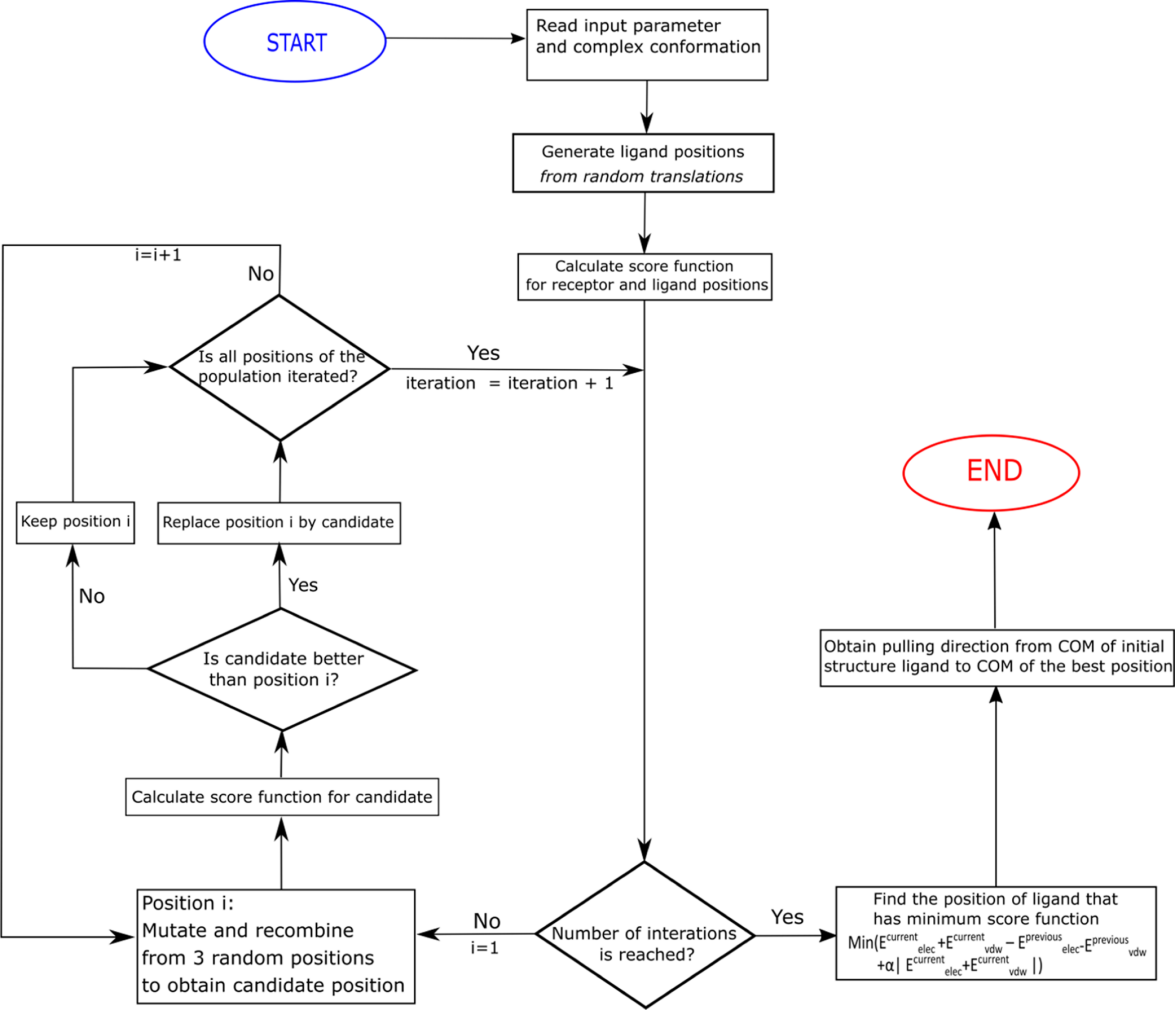
Main steps in the novel protocol for obtaining the optimal zigzag pulling pathway. COM refers to the center of mass of the ligand.

Let us describe the DE^71^ algorithm in more detail. This algorithm is an optimization method used to find the minimum of an objective function that is a D-dimensional real-valued function. It directly searches the optimal solution from the objective function, which does not require gradient information. The main steps of DE are described in Figure 4. First, a set of ligand positions are generated by random translations (Figure 3B). For the first step shown in Figure 3A, the ligand conformations obtained at the end of the *NPT* simulation were used for the DE algorithm. However, for the remaining short intervals, we used the ligand conformations generated in the SMD simulation to perform the optimization using DE.

The number of positions and the magnitude of the translation vector can be set by the user, but we can show that 3000 ligand positions and the vector length of 7 Å are a reasonable choice. Figure S5 shows the effect of the number of conformations on the pulling direction of ligand 10 in LSD1. Our result indicates that the pulling direction is converged at 2000 conformations. Therefore, our choice of 3000 is safe. Figure S6 shows the dependence of the pulling direction obtained by using DE with 3000 ligand conformations versus the vector length for ligand 10 interacting with LSD1. It is obvious that the pulling direction is converged at a length of 7 Å. Hence, this value guarantees locality and is not too small to allow the ligand to wobble around its original structure. If the vector length is too long, the direction may point through the protein into the outer space because the potential value at that position is small, and this is what we want to avoid.

The DE algorithm generates new candidate solutions by adding a weighted difference between the two candidates to the third candidate, and the new candidate is called a mutation. This mutation is mixed with the parameters (crossover) of another candidate (target candidate), resulting in a trial candidate. If the trial candidate has a lower value of objective function than the target candidate, the trial candidate replaces the target candidate in the population. This process is iterated until the termination criterion is met (Figure 4). To find out how many iterations are sufficient to obtain reasonable results, we examined the dependence of the pulling direction on the number of iteration steps for ligand 10 leaving LSD1 (Figure S7). After 25 and 50 iterations, the pulling direction does not converge. However, when the number of iterations is equal or greater than 100, the result converges. Therefore, we chose 100 as the number of iterations in this work to balance performance and reliability.

In short, based on the DE algorithm,^71^ the ligand positions are refined for the best score function value at each iteration (see eq 3 for the score function). In this work, the cross probability (CR) and differential weight (*F*) of the DE algorithm were chosen as 0.9 and 0.5, respectively, that is, we used the same set of parameters as those in the original paper of Storn and Price.^72^ After 100 iterations, the positions with the smallest score function are selected to find the next step of the pulling direction (Figure 3A). As soon as the ligand leaves the protein, the pulling direction is kept unchanged.

As mentioned above, for each small step (Figure 3A), after DE, we performed a 20 ps SMD simulation at a pulling speed *v* = 5 nm/ns, where the ligand was allowed to be flexible. To show that the choice of the time interval τ = 20 ps is reasonable, we tested the time intervals τ = 10, 20, and 40 ps for SMD simulation for the HupA–TcAchE complex. A typical force–time profile for one trajectory is shown in Figure S8. We excluded τ = 40 ps because there is a sharp change after passing the peak (see the black box at the bottom). Since the rupture forces are similar for τ = 10 and 20 ps, we chose 20 ps to save simulation time. The user can use shorter intervals for smoother interval switching change between intervals.

Finally, for the convenience of the user, we summarize the parameters used in our method in Table 1. We recommend this set of parameters, but the user can try other sets as well.

**Table 1:** Parameters Used in Our Method

name	value
number of populations (random vectors) (see Figure 3B)	3000
maximum length of random vectors	7 Å
number of iterations in DE for each step (see Figure 3A)	100
pulling speed *v*	5 nm/ns
time interval τ	20 ps
α (see eq 3)	2
CR	0.9
differential weight (*F*)	0.5

### Our Protocol Can Reproduce Two Previously Known Exit Paths for HupA from TcAChE

**Figure 5:**
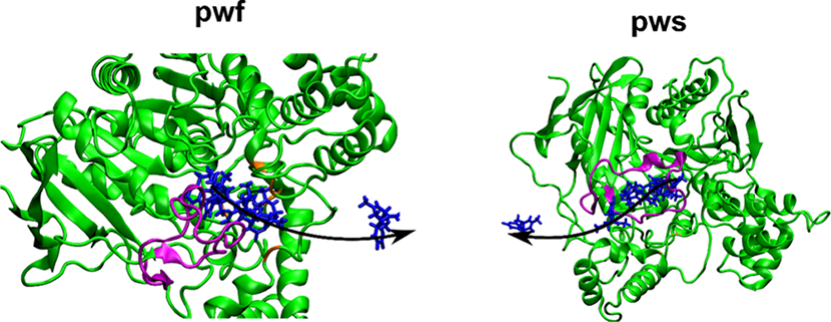
Two pathways of HupA to exit from the TcAche binding cite. The result obtained in this work coincides with the paths previously reported by other groups.^72,95,96^ Snapshots of the ligand are shown in blue licorice, while the Ω-loop is shown in purple.

Our protocol was aimed at solving problems with pulling of ligands from LSD1, but we must first check its reliability for systems with known unbinding pathways. Previous computational studies^72,95,96^ have identified two dissociation pathways for HupA from the TcAChE binding site (Figure 5). The first pathway is called the front exit pwf, and the second is the transiently opened side exit pws.

Pwf is a tunnel from the CAS (catalytic anionic site) of TcAChE that involves residues Y70, V71, D72, Y121, W279, F290, F331, and Y334. There is no rearrangement of TcAChE residues, which is required for the HupA escape along this pathway. The second pathway pws from the CAS to the solvent is formed by a transient channel via the Ω-loop, which comprises residues 67–94 (Figure 5). The research by Tara et al. suggested that pws can play as a channel for water and ion molecules in the HupA-bound structure.^96^ In the simulations carried out by Rydzewski et al.,^72^ HupA can leave through pws, but the Ω-loop opening is needed.^72^ Due to the presence of the transient channel that includes the Ω-loop opening, the ability to capture the two dissociation pathways of HupA can be considered a benchmark for our protocol.

Using our protocol, we have also obtained two pathways, which coincide with previously identified pwf and pws^72^ (Figure 5), which means that the protocol based on DE is reliable in determining dissociation pathways of the TcAChE–HupA complex.

In our simulations, HupA exits from the binding pocket via pwf and pws pathways in 14 and 6 SMD trajectories, respectively. This observation is understandable, and since there is a structure restructuring to open the Ω loop, it takes a while for pws to excute whereas pwf is already open, which forces HupA to favor pwf over pws. To further support this result, we show the time dependence of the root mean square deviation (RMSD) of the Ω-loop in two pathways (Figure S9). For pwf, we have a monotonic dependence, while for pws, a sudden change at about 1650 ps indicates the opening of the Ω-loop.

The average simulation times to determine the exit channels are about 1636 and 1989 ps for channels 1 and 2, respectively (Table S4), which is less than the cutoff value of 3000 ps (Table S3). This is because, as mentioned above, the search process usually ends before the maximum time is reached.

We calculated the nonbonded interaction energy between HupA and TcAChE for two pathways (Figure S9). Since the simulation is terminated when HupA is pulled out of TcAChE, but they are not far away, interactions are still present at the end of the simulation. In both cases, the interaction energy changes sharply when HupA leaves TcAChE, but the change in the pwf case is softer, which is consistent with the Ω-loop opening in pws.

Since the difference in the interaction energies between the initial and final states in pws is smaller than in pwf (Figure S9), we anticipate that although pws requires the Ω-loop opening, the potential barrier is lower along this pathway. Therefore, opening of the Ω-loop lowers the potential barrier, indicating that both these pathways are feasible. In addition, we did not observe any evidence that HupA leaves TcAChE via the so-called backdoor pathway (pwb) that was reported for other ligands.^97^ This result shows that pwb is not an HupA dissociation pathway, which is consistent with Rydzewski et al.^72^

### Our Protocol Can Identify Five Previously Known Exit Paths for Camphor from P450cam

It is already known that camphor can escape from the P450cam binding site via five major pathways,^53,62^ making the problem more difficult than in the HupA case.^98^ Because the interior of the P450cam molecule is complex, we performed 60 independent SMD trajectories to determine the exit paths for camphor using our protocol. For comparison, we used the same structural nomenclature of P450cam as Poulos et al.^77^ and Rydzewski and Nowak^62^ P450cam comprises 13 helixes labeled A, B, B′, and C-L and five β sheets labeled 1–5 (Figure S10).

**Figure 6:**
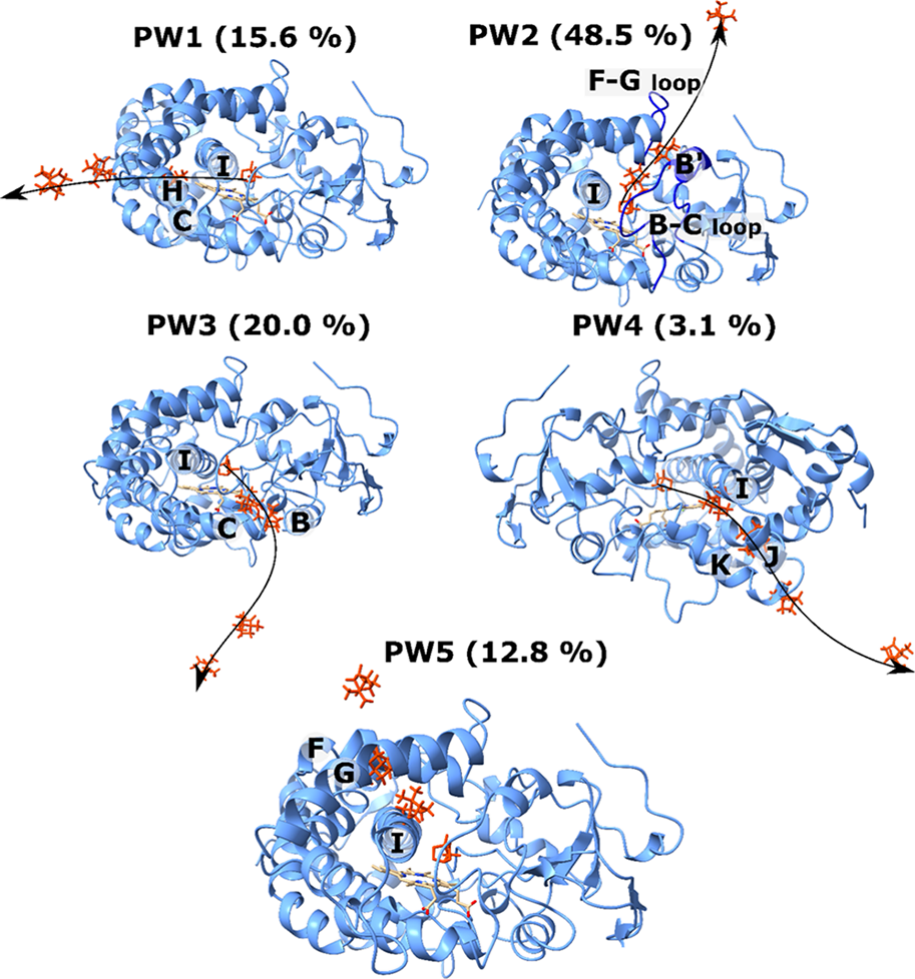
Schematics for dissociation pathways of camphor from P450cam. The arrows represent pathways obtained from this work. Several snapshots of ligands are shown in orange licorice. HEM group is shown in licorice. The number in brackets represents the population of the pathway obtained from bootstrap analysis.

Camphor passes between helices I and C in the first path (PW1) (Figure 6), which depends on the flexibility of spiral C. This path has been discovered from previous simulations using RAMD and thermal motion pathway methods,^53,99,100^ as well as using the machine learning-based dimensionality reduction method.^62^

The second pathway (PW2) starts from the L244-G248 residues of helix I and passes between the B′ helix (residues E91–A92) and the F–G loop (R186–F193) (Figure 6).^62,99,101^ It has been suggested to subclass this path, as in the RAMD simulations,^53^ but Rydzewski and Nowak^62^ found only one class that is the most likely.

The third pathway (PW3) includes helix I and B–C loop and passes between helices B and C including residues Y75–H80 and D104–P106, which is believed to be the channel for 5-hydroxyl-camphor.^77,102^ This pathway was also found by Rydzewski and Nowak^62^ Previous research studies suggest that this pathway may play a role in the electron transport or water network for proton transport.^99,103^ The fourth pathway (PW4) was identified in a study by Rydzewski and Nowak,^63^ which is adjacent to helix I and passes helices J and K (E273–R280). This pathway is not observed in previous RAMD studies. The fifth pathway (PW5) was found in the RAMD simulation,^53^ which is called pathway class 3. However, this pathway was not observed by Rydzewski and Nowak.^62^ The ligand traverses helix I at residues L244–G248 and then crosses the space between helices F (I174–D182) and G (A199–L204) (Figure 6).

Using our protocol and 60 SMD trajectories, we obtained five major egress pathways of camphor, of which the first four pathways are the same as those indicated by Rydzewski and Nowak^63^ (Figure 6), while PW5 is similar to pathway class 3 in RAMD simulations.^53^ PW1–PW5 occurred in 11, 31, 9, 2, and 7 trajectories, respectively. By performing a bootstrap analysis with the size of the resampled data set of 20,000, we can show that the average populations are 15.6 ± 3.4, 48.5 ± 5.1, 20.0 ± 4.1, 3.1 ± 0.8, and 12.8 ± 3.7 for paths 1, 2, 3, 4, and 5, respectively. Thus, in line with the previous work, we obtained the most probable PW2 with a population of 48.5% but not the subclasses pathways identified in RAMD simulations.^53^

PW4 has the lowest probability (2/60 ≈ 3%), which is consistent with Rydzewski and Nowak. PW5 (≈12%) is more likely than PW4, and this pathway was observed in RAMD simulations^53^ but not in Rydzewski and Nowak.^62^ In RAMD simulations, PW5 has the least population, which agrees with our result.

**Figure 7:**
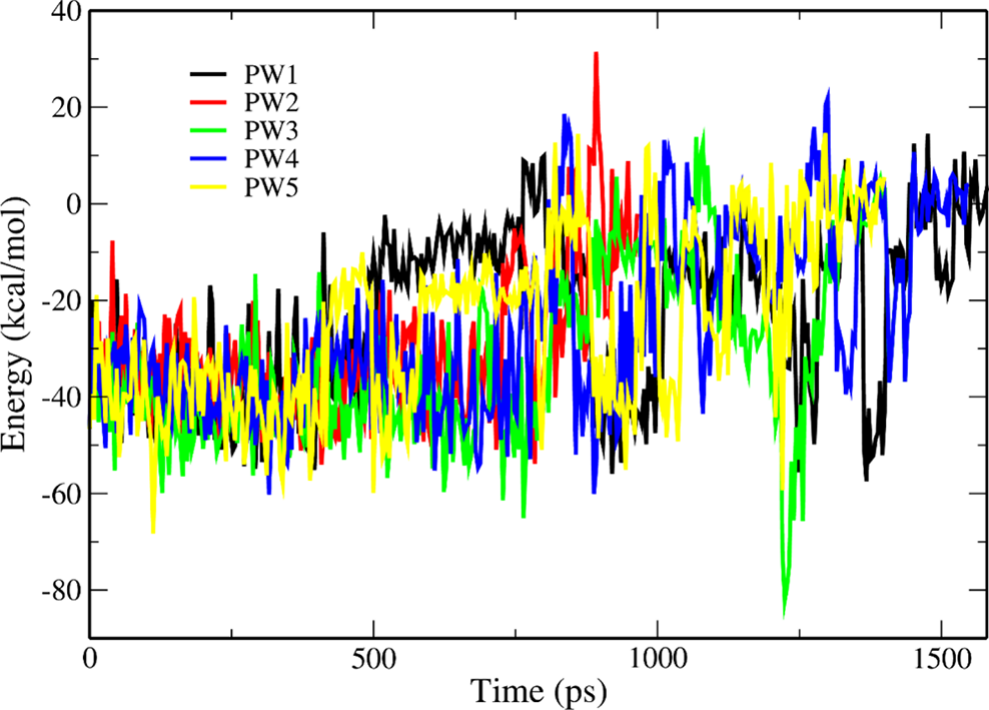
Time dependence of the energy of interaction between P450cam and camphor along five pathways.

From the time dependence of the nonbonded interaction energy (Figure 7), it is clear that the ligand takes the shortest time to escape from P450cam along PW2 (see also Table S5), which is consistent with the fact that PW2 is the most probable route. In addition, *W*_pull_ is the smallest in PW2 (Table 2), which also confirms this observation. However, *W*_pull_ in PW1 is larger than in PW3 and PW5, which contradicts the fact that the population of PW1 is higher than that of PW3 and PW5. This may be due to the fact that the number of SMD runs is not enough to get a reliable result. Overall, our protocol is robust because it can predict the same pathways as those in previous studies for both P450cam-camphor and TcAChE–HupA complexes.

**Table 2:** Pulling Work (kcal/mol) of Camphor Obtained in Five Unbinding Pathways^a^

PW1	PW2	PW3	PW4	PW5
254.47 ± 26.41	128.97 ± 5.71	169.06 ± 14.24	389.42 ± 46.77	152.51 ± 13.99

a The error represents the standard deviation.

### Zigzag Pulling Improves the Performance of SMD Simulation for LSD1

As mentioned above, in the case of LSD1, unidirectional pulling led to a second peak in the force–time/extension profile. This artifact arises from the collision of the ligand with the surface of the narrow exit tunnel, which affects the rupture force and pulling work, resulting in a low correlation between the theory and experiment. To solve this problem, instead of pulling with a constant direction, we took a multidirectional approach. Since our DE-based protocol works well for the HupA–TcAChE and P450cam-camphor systems, we used it to study the binding affinity of 24 ligands for LSD1.

**Figure 8:**
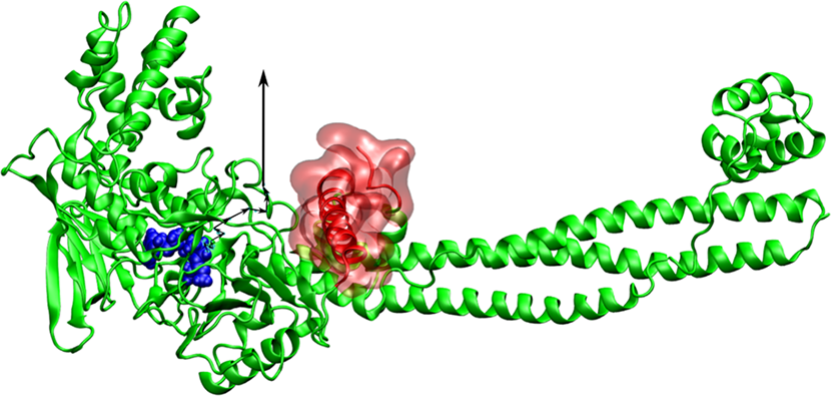
Schematic for the zigzag pulling direction (black arow) of the ligand (blue) from LSD1. The obstacle region is show in red with a transparent surface.

**Figure 9:**
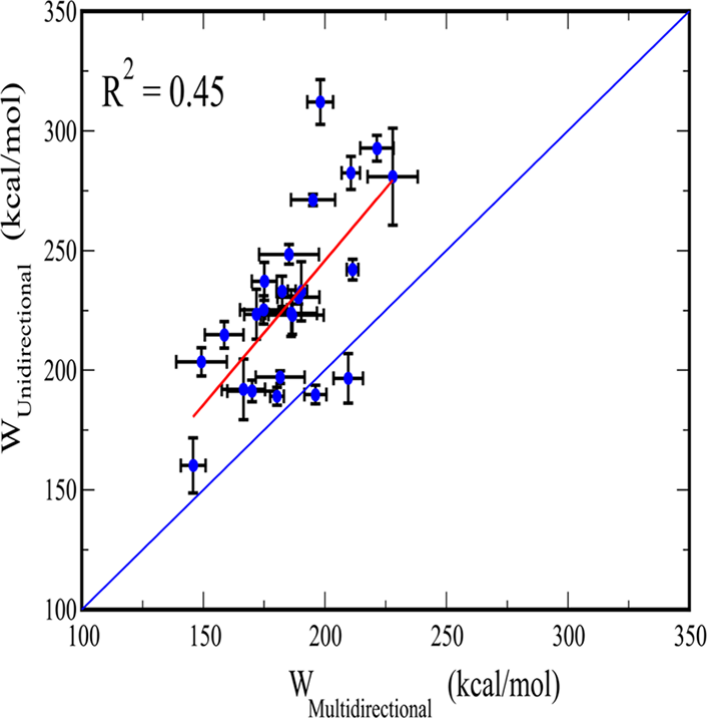
Pulling work obtained using SMD simulations with unidirectional and multidirectional pulling for 24 ligands bound to LSD1. The red line is a linear fit of the two data sets with a correlation level *R*^2^ = 0.45. The results were obtained at *v* = 5 nm/ns and averaged over 20 SMD trajectories, and error bars represent standard deviations.

In SMD simulations, the pulling direction is determined on the fly, and the initial conformations as well as other setup parameters are the same as those in the straight pulling. The multidirectional pathway avoids the region where the collision between the ligand and the receptor occurs in unidirectional stretching (Figure 8). Figure S11 shows typical force–time profiles, obtained with straight and zigzag pulling. Clearly, the second peak is much more pronounced in the unidirectional case, which implies that the multidirectional path avoids collision between the ligand and the receptor. In accordance with this observation, *W*_pull_ performed by the ligand during the exit along the zigzag path is lower than in the forward case (Figure 9). Correlation between work values obtained using two pulling methods is *R*^2^ = 0.45.

**Figure 10:**
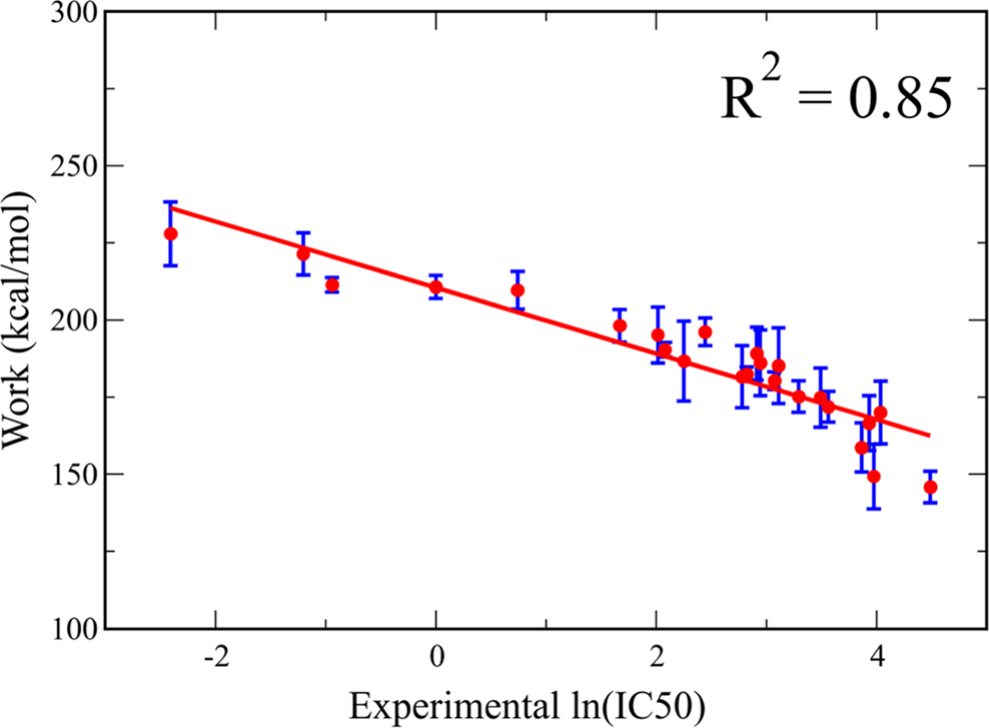
Pulling work as a function of the experimental ln(IC50) with IC50 measured in moles per liter. Results were obtained using SMD simulations, in which the DE-based protocol was employed to identify the zigzag pulling direction. The red line represents the data fit function. *R*^2^ is the correlation coefficient. The results were obtained at *v* = 5 nm/ns and averaged over 20 SMD trajectories, and error bars represent standard deviations.

The correlation between *W*_pull_ and experimental ln(IC50) is *R*^2^ = 0.85 (Figure 10), which is better than *R*^2^ = 0.43, obtained in SMD simulations using a constant pulling direction. The improved correlation between the theory and experiment proves the superiority of the new protocol.

It should be noted that the correlation level *R*^2^ = 0.85 (Figure 10) was obtained at *v* = 5 nm/ns. In order to show that the result is independent of the pulling speed, we ran simulations at *v* = 1 nm/ns and obtained *R*^2^ = 0.86 (Figure S12), which is very close to 0.85. Thus, as in previous studies,^48^ the correlation between *W*_pull_ and experimental ln(IC50) is robust against *v*.

### Finding Zigzag Paths Using the New Protocol Does Not Take Much Time

When simulating the TcAChE–HupA and P450cam-camphor complexes, there is no trajectory along which ligands get stuck inside the receptor, which shows that our protocol has 100% success. From this point of view, our method is better than RAMD, which has a success rate below 100%.^53,104^

The simulation time to determine the five exit pathways of camphor out of P450cam is about 1000 ps (Table S5), which is much higher than the RAMD time spent on pathways 1, 2, and 5.^53^ Since both methods used almost the same number of trajectories, our method is more than an order of magnitude slower than RAMD. Overall, however, we can improve performance by increasing the pulling speed. Although our method is slower than RAMD, the computation time required to find the egress channels is much less than the total time. Using a node of two CPUs, Intel(R) Xeon(R) Gold 6136 CPU @ 3.00GHz with 12 physical cores per CPU, and four GPUs, NVIDIA Tesla V100-SXM2-32GB, our protocol consumes less than 3% of the total computational time for LSD1, TcAChE, and P450cam targets, indicating that the DE-based algorithm is not time-consuming and is effective in finding release paths in complex systems. For example, the simulation time is about 8 h for 10 trajectories of LSD1–ligand complexes, and the wall clock time is about 8 h 12 min.

### Is the Dissociation Pathway Reversible?

In the previous section, we identified the ligand dissociation pathways for several complexes. An interesting question emerges: is the dissociation pathway reversible? Since the *E*_score_ function only has a term that prevents a ligand from being energetically trapped, but no assumptions have been made about the unbinding or binding process, our protocol must be applied to determine the association pathways. To test the reversibility of the pathway, we tried to find a pathway for HupA to associate with TcAChE, which begins with the last snapshot of the dissociation pathway. Out of five attempts, only in one case (20%) does the ligand come back to the binding site following the same unbinding pathway. Although the success rate is not high, this demonstrates that, as a proof of concept, our protocol works for ligand–protein association. However, the solution of the problem of reversibility of the dissociation path using our approach with different sets of parameters requires further research because verifying whether this method can be used to compute the ligand association, as we have done here, and determining whether the dissociation paths are reversible are generally different problems.^105^

## Conclusions

Using SMD simulations with a straight pulling direction, we cannot properly describe the ligand release from LSD1 as the correlation between the simulated pulling work and experimental data on IC50 is quite low. This is due to the narrow tunnel, which leads to the collision of the ligand with some residues of the receptor. To overcome this difficulty, we proposed a new approach for defining a multidirectional pulling path and used it for SMD simulations instead of a unidirectional path. In our protocol, the release pathway is derived from a minimum of receptor–ligand interaction using a DE algorithm. We have shown that the protocol is successful in identifying pathways previously obtained using other methods for the TcAChE–HupA and P450cam–camphor complexes.

By applying the new method to study the binding affinity of 24 ligands to LSD1, we obtained a much better agreement with the experiment compared to the direct exit pathway SMD simulation. It would be useful to check the reliability of our protocol for other systems.

Recently, it was shown that similar to SMD,^48^ TMD can be used to discern binding affinities of small compounds based on the non-equilibrium work.^19^ In TMD, the ligand position can be changed as long as the distance (reaction coordinate) is maintained by the constraint. This differs from SMD, where the ligand movement is navigated by the direction of the pulling force. Hence, in SMD, the pulling direction is important, while in TMD, the constraint is crucial. In the case of the unidirectional pathway, TMD and SMD are very similar because the straight pulling direction can also be a distance constraint. However, in complex systems such as LSD1, in SMD, the zigzag pulling direction must be determined in order to steer the ligand out from the binding site, while in TMD, a reasonable constraint is needed to obtain the ejection pathways. Since the pulling direction and constraint can be different, it would be interesting to know if it is possible to use TMD instead of SMD in our protocol to find multidirectional paths. Work in this direction is in progress.

## Supporting Information Available

CID, name, and IC50 of the ligands studied in this work; grid size and grid center used in docking simulation for all targets; simulation time τ_sim_ for each SMD trajectory; simulation time for determining egress pathways of HupA from TcAChE; simulation time (ps) for determining exit pathways of camphor from P450cam; initial structure for SMD simulations of LSD1 and ligands obtained from docking simulation; work profiles obtained for 24 ligands bound to LSD1; dependence of the distances between the COM of ligand 1 and regions R308–G313 and K371–F396 of LSD1, force experienced by ligand 1, and the interaction energy between ligand 1 and regions R308–G313 and K371–F396 of LSD1 on the displacement; the pulling directions obtained using the MSH algorithm for LSD1; Figure S5: dependence of the pulling direction on the number of positions for ligand 10 interacting with LSD1; dependence of the pulling direction on the vector length for ligand 10 interacting with LSD1; dependence of the pulling direction on the number of iterations; force–time profiles obtained using SMD for the HupA–TcAchE complex for τ = 10, 20, and 40 ps; time dependence of nonbonded interaction energies of HupA and TcAchE along pathways pwf and pws and of RMSD of the Ω-loop; structure of cytochrome P450cam; force–time profiles obtained with straight and zigzag pulling for LSD1; and pulling work obtained at *v* = 1 nm/ns as a function of the experimental ln(IC50) (PDF)
Main code and scripts used to run simulations (ZIP)

## Notes

Data and software availability: the initial and representative structures of the two exit pathways of HupA from TcAChE are presented in the PW_TcAChe.zip file. The PW_P450cam.zip file contains the initial and representative structures of the five pathways to expel camphor from P450cam. The initial and representative structures of ligand 1 in complex with LSD1, obtained in a multidirectional trajectory, are stored in the LSD1_lig1.zip file. Our main code in C++ and bash script files for running simulations to determine unbinding paths are available in the “Code-Scripts.zip” file. The AutoDockTools and AutoDock Vina, used for docking simulations are available free of charge at http://autodock.scripps.edu/resources/adt and http://vina.scripps.edu, respectively. The AmberTools, which consists of Antechamber, can be downloaded for free at https://ambermd.org/AmberTools.php. The Acpype tool was downloaded from https://github.com/alanwilter/acpype. The GROMACS 2018 package was obtained from https://manual.gromacs.org. 3D visualization was performed by using the Pymol package and VMD software, which can be obtained from https://pymol.org/2/ and https://www.ks.uiuc.edu/Research/vmd/, respectively. Qtgrace, used for plotting, can be downloaded from https://sourceforge.net/projects/qtgrace/. Instructions for using our code are available in the README.txt file.
