# Supplementary material for: Determination of Multidirectional Pathways for Ligand Release from the Receptor: A New Approach Based on Differential Evolution
Source: J Chem Theory Comput. 2022 May 5;18(6):3860–72. doi: 10.1021/acs.jctc.1c01158 (PMC9202309; doi:10.1021/acs.jctc.1c01158)
Supplement: Supplementary file 1 — ct1c01158_si_001.pdf [file ct1c01158_si_001.pdf]

Determination of Multidirectional Pathways for Ligand Release from the Receptor: A New Approach Based on Differential Evolution

Hoang Linh Nguyen[1] ,Nguyen Quoc Thai[1,2], and Mai Suan Li[3,*]

[1]Life Science Lab, Institute for Computational Science and Technology, QuangTrung Software City, Tan Chanh Hiep Ward, District 12, Ho Chi Minh City, Vietnam

[2]Dong Thap University, 783 Pham Huu Lau Street, Ward 6, Cao Lanh City, Dong Thap, Vietnam

[3]Institute of Physics, Polish Academy of Sciences, al. Lotnikow 32/46, 02-668, Warsaw, Poland

*Email: masli@ifpan.edu.pl

SUPPORTING INFORMATION

Table S1: CID, name, 2D structure and IC50 of the ligands studied in this work for the LSD1 target. The *in vitro* IC50 was obtained using the mass spectrometry (MS) method, time-resolved fluorescence resonance energy transfer (TR-FRET) assays, the homogenous time resolved fluorescence (HTRF) assay, peroxidase assay, and MTT assay.

No.	CID	Ligand	2D structure	IC_{50} (μM)	Ref
1	13175575	Polyamine 7	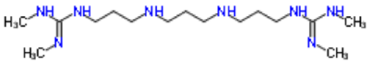	1.0	1
2	34449629	CBB-1007	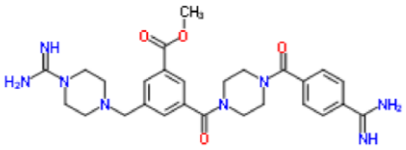	5.3	2
3	624502	Namoline	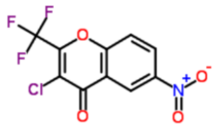	51.0	3
4	28667770	Amidoximes	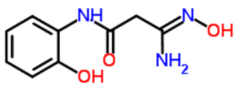	16.8	4
5	71662892	Phenyl oxazole 12a	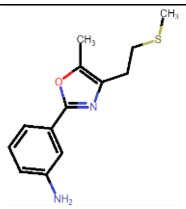	16.1	5
6	31104030	Phenyl oxazole 13a	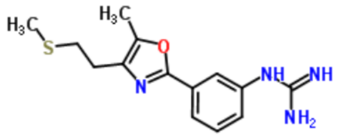	9.5	5
7	31104078	GSK-354	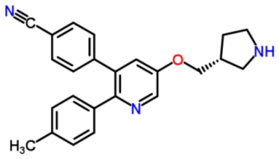	0.09	6,7
8	26755567	Aminothiazole 16	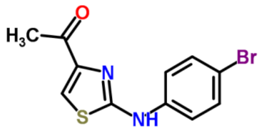	26.9	7

9	31104049	Aminothiazole 17	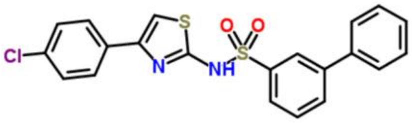	7.5	7
10	31125416	Huntsman	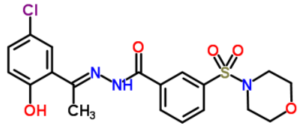	0.013	8, 9
11	29414493	Dithiocarbamate 19a	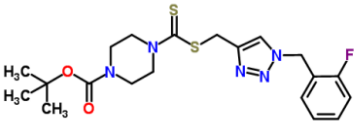	8.0	
12	29419602	Dithiocarbamate 19b	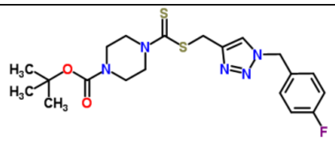	21.6	
13	29417948	Dithiocarbamate 19c	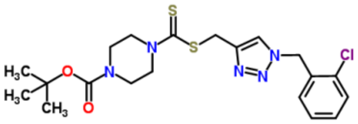	11.5	
14	29415686	Dithiocarbamate 19d	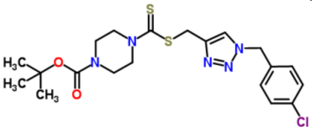	56.5	
15	29414835	Dithiocarbamate 19e	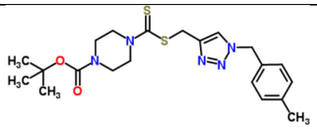	2.1	10, 11
16	29417630	Dithiocarbamate 19f	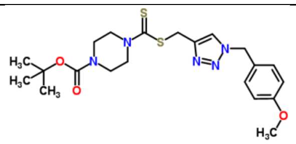	18.4	
17	29416415	Dithiocarbamate 19g	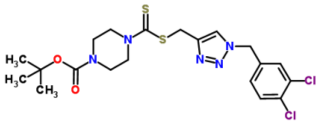	88.9	
18	29415409	Dithiocarbamate 19h	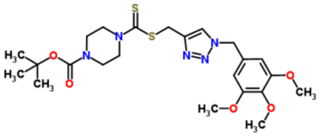	19.0	
19	29417544	Dithiocarbamate 20a	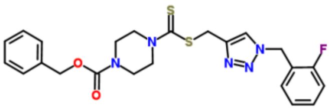	32.8	

20	29416299	Dithiocarbamate 20b	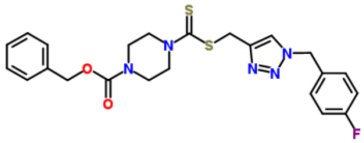	47.6	
21	29419119	Dithiocarbamate 20c	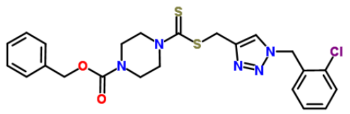	53.2	
22	29420288	Dithiocarbamate 20e	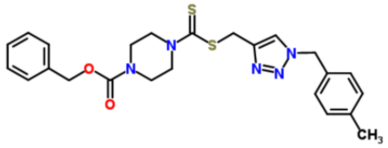	22.4	
23	29419510	Dithiocarbamate 20f	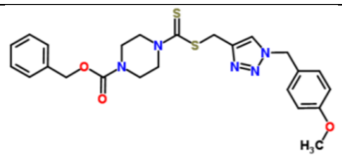	35.2	
24	29418220	Dithiocarbamate 21	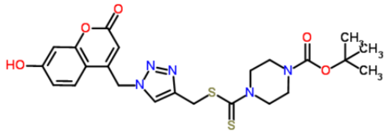	0.39	

Table S2. Grid size and grid center used in docking simulation for all targets.

Target	Grid size (Å)	Grid center (Å)
LDS1 (PDB ID: 2UXN)	30x40x30	66.62, 65.75, 30.68
TcAChE (PDB ID: 1AE5)	60x60x65	10.44, 18.78, 32.20
P450cam (PDB ID: 2CPP)	30x30x35	47.12, 45.49, 10.08

Table S3. Simulation time τ_{sim} for each SMD trajectory. The number of steps N used in the search for egress pathways (Figure 3A), $N = \tau_{sim}/\tau$, where interval τ = 20 ps.

Parameter	LSD1	TcAChE	P450cam
Simulation time for each trajectory	1000 ps	3000 ps	1540 ps
Number of steps N (see Fig. 3A)	50	150	77
Number of trajectories	20	20	60

Table S4. Simulation time for determining egress pathways of HupA from TcAChE. The result was averaged over 14 and 6 successful trajectories for pathways 1 and 2, respectively.

Pathway	Time (ps)
1 (pwf)	1636.0 ± 51.8
2 (pws)	1989.3 ± 102.3

Table S5. Simulation time (ps) for determining exit pathways of camphor from P450cam. The result was averaged over successful trajectories as described in the main text. For comparison, we show the simulation time per trajectory (14 trajectories in total) used in RAMD[51] (Lüdemann et al, J Mol. Biol. **2000**, 303, 797-811).

Pathway	Our method<br>Time averaged over trajectories	RAMD<br>Simulation time per trajectory
1	1290.0 ± 9.0	20
2	1060.0 ± 55.4	10-190
3	1345 ± 67.8	Not found
4	1460.0 ± 22.0	Not found
5	1180.0 ± 62.3	20-50

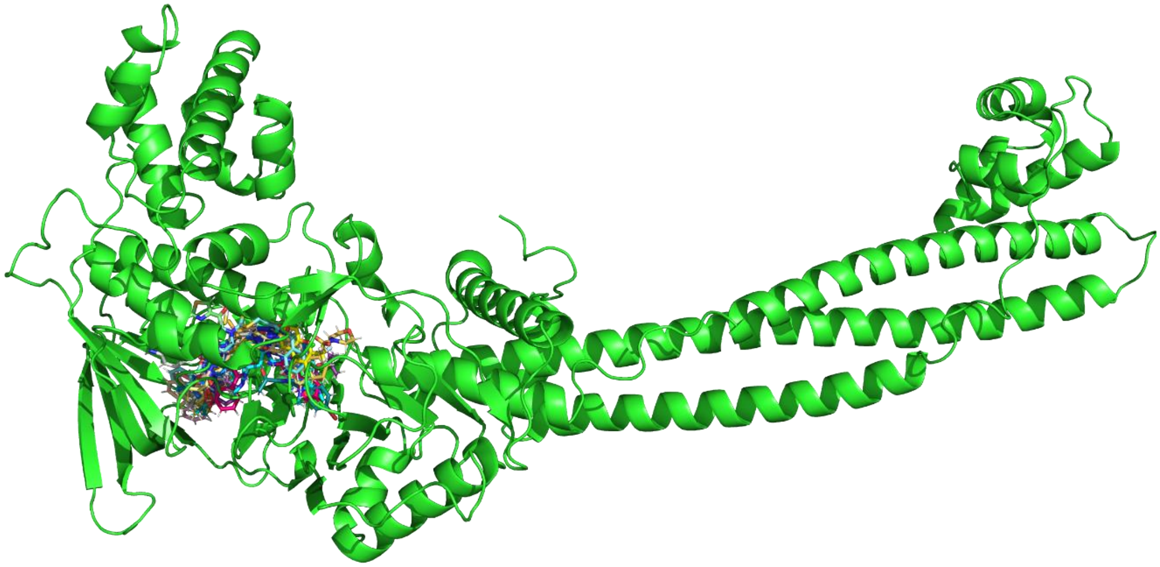

Figure S1: The initial structure for SMD simulations of LSD1 and ligands obtained from docking simulation. The protein is in green cartoon and ligands are in sticks.

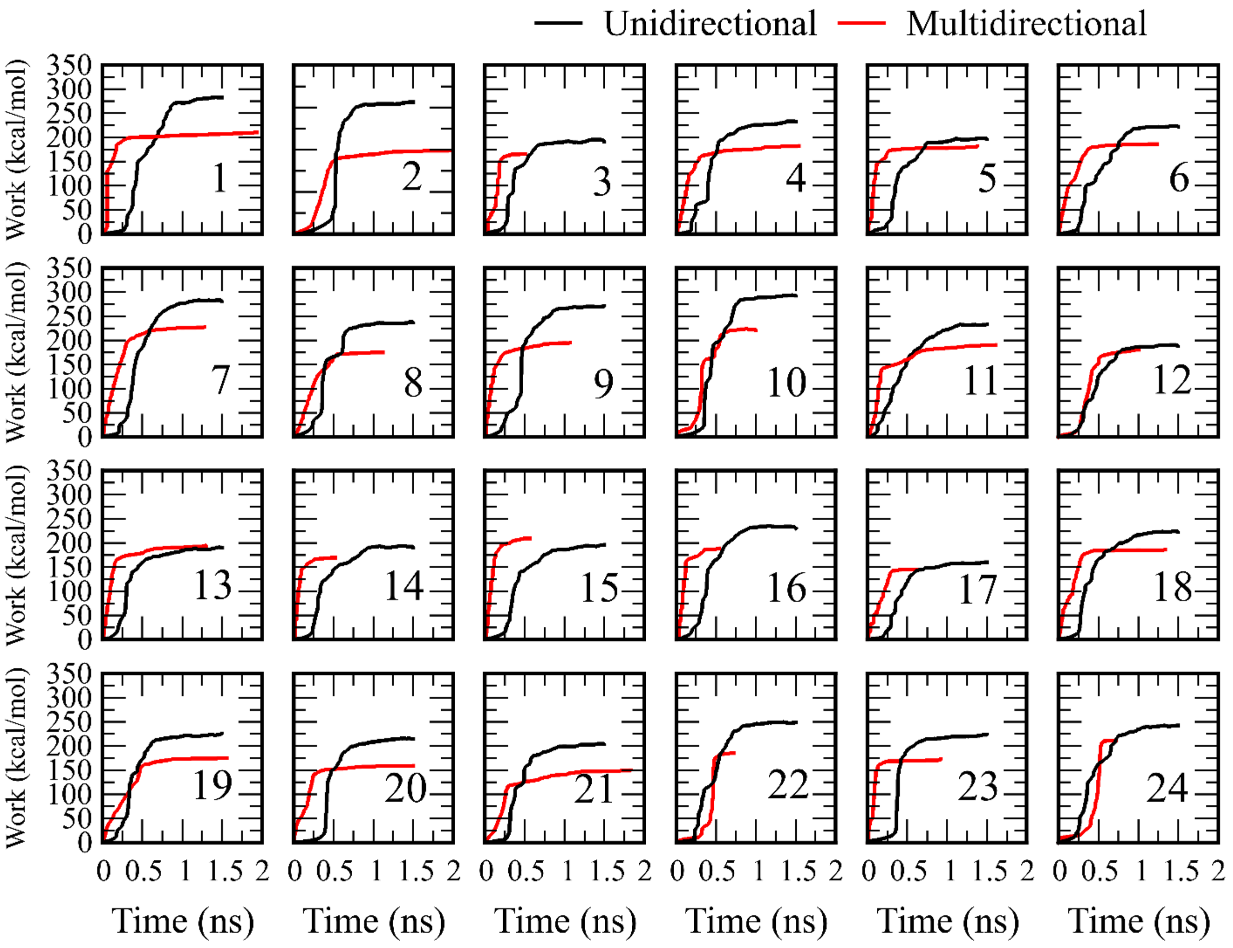

Figure S2. Work profiles obtained for 24 ligands bound to LSD1 using unidirectional (black) and multidirectional (red) pulling. The number is the ligand number shown in Table S1. W_{pull} was defined as work at the last point. Results were averaged over 20 SMD trajectories. Note that the simulation time depends on the ligand and pulling path, because the simulation stops when the ligand leaves the protein and goes deep into the solution.

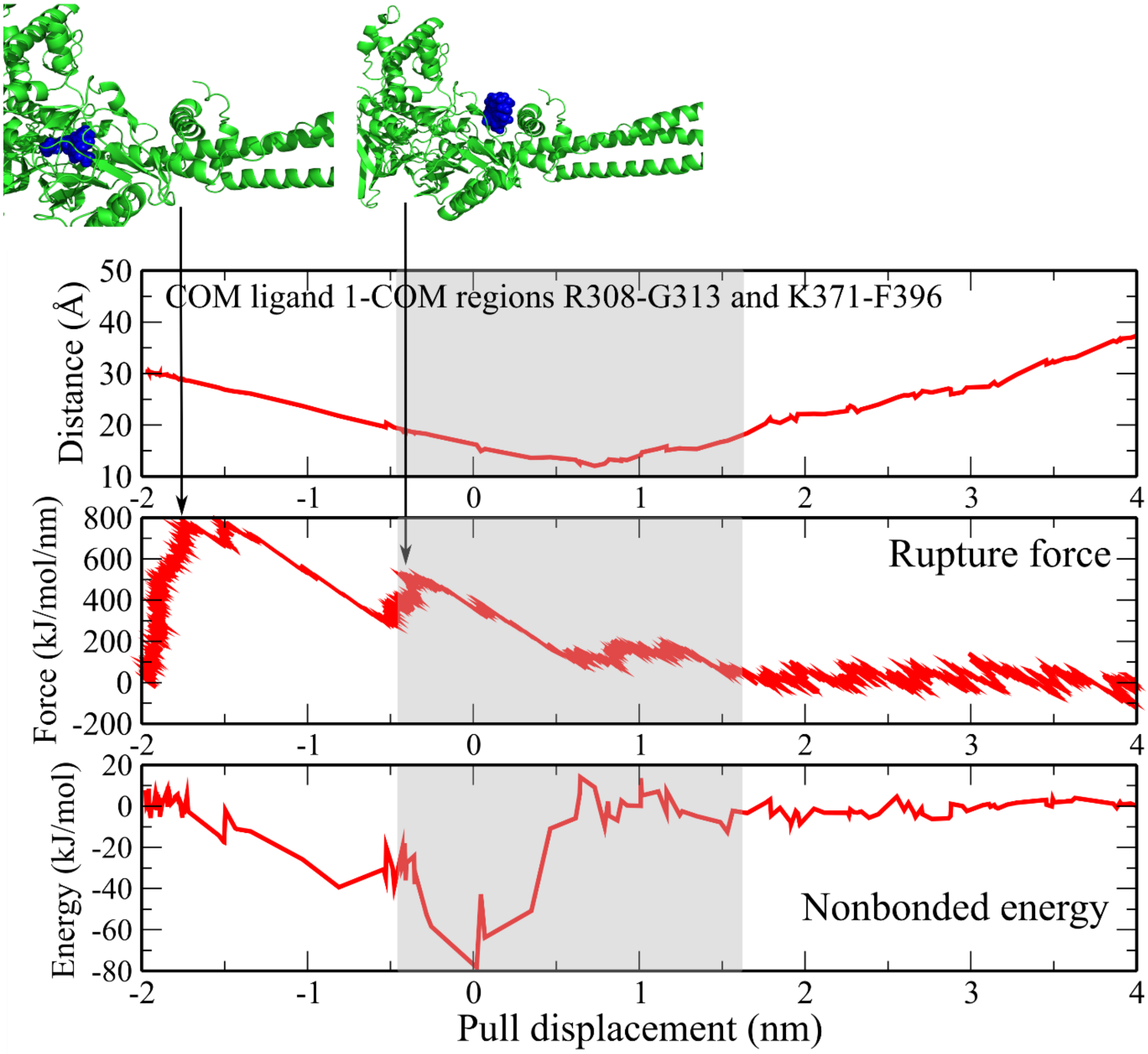

Figure S3: (Upper part) Distances between COM of ligand 1 and regions R308-G313 and K371-F396 of LSD1. The result was obtained in a typical SMD trajectory with unidirectional pulling for ligand 1 (Polyamine 7). (Middle part) Force-displacement of ligand 1. (Lower part) nonbonded interaction energy between ligand 1 and regions R308-G313 and K371-F396 of LSD1. Light gray rectangles represent the time interval during which ligand 1 approaches the R308-G313 and K371-F396 regions of LSD1.

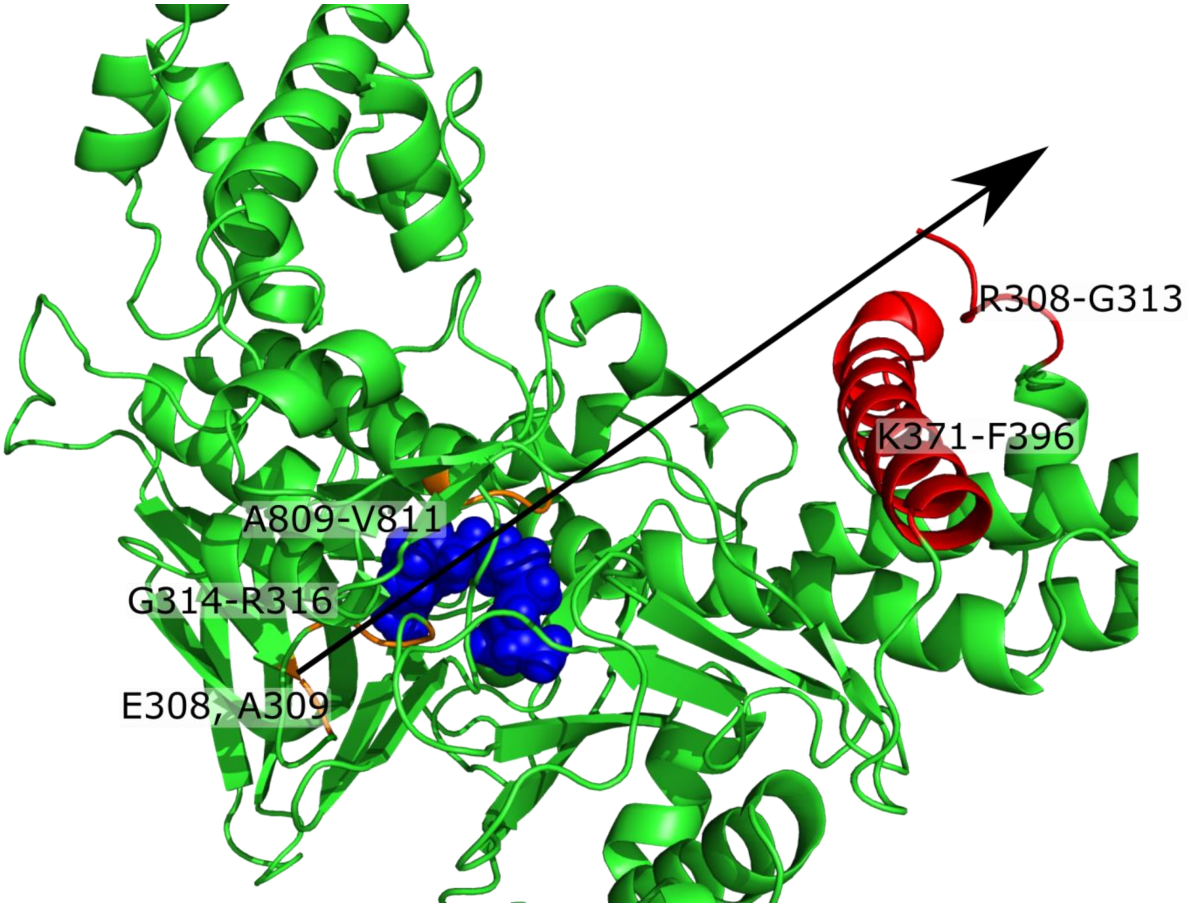

Figure S4: The pulling directions obtained by the MSH algorithm is a vector connecting E308-A309 with G314-R316 and A809-V811 regions of chain A and then directed to a location next to R308-G313 and K371-F396 of LSD1 of chain B. Green and red refer to chain A and B, respectively.

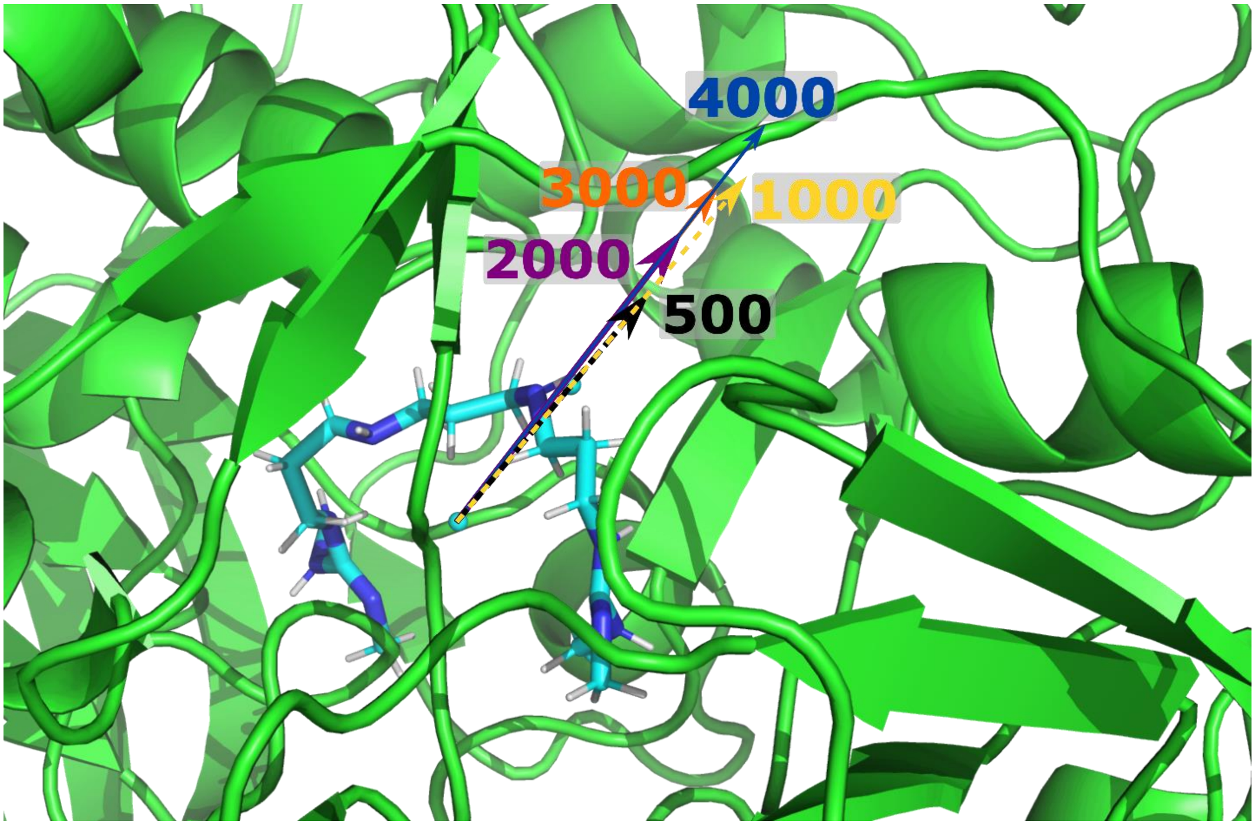

Figure S5: Dependence of the pulling direction on the number of positions for ligand 10 interacting with LSD1. The result was obtained by DE with a vector length of 7 Å.

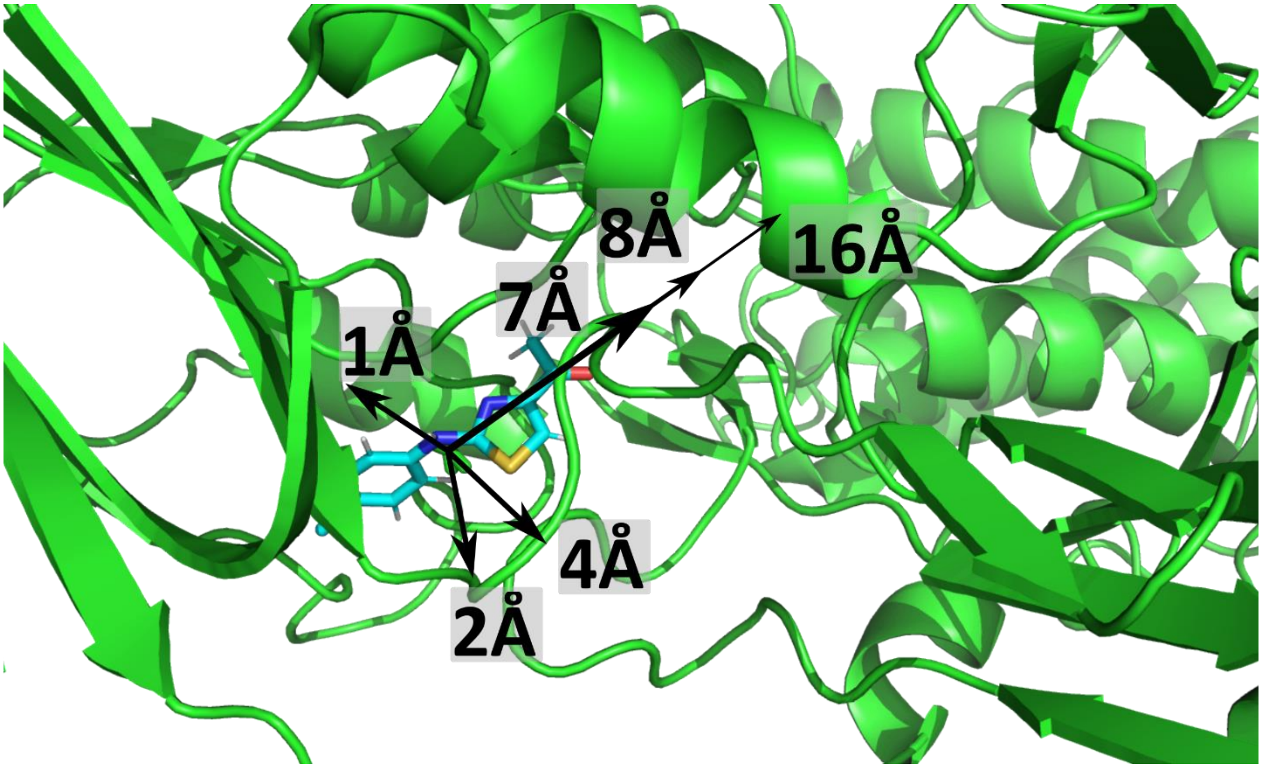

Figure S6. Dependence of the pulling direction on the vector length for ligand 10 interacting with LSD1. The result was obtained by DE with the number of ligand positions of 3000.

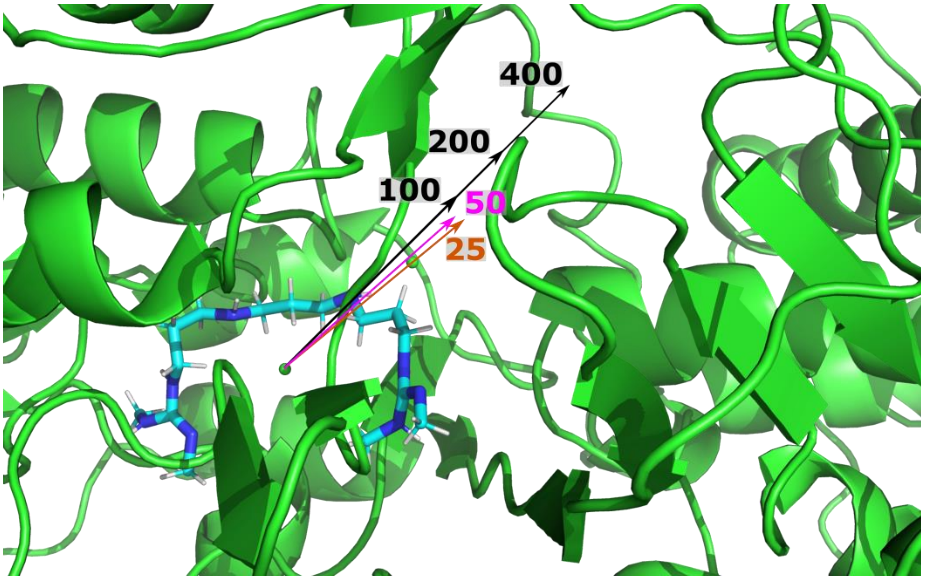

Figure S7: Dependence of the pulling direction on the number of iterations. The result was obtained for ligand 10 in LSD1using 3000 initial random positions and the maximum translation distance of 7Å.

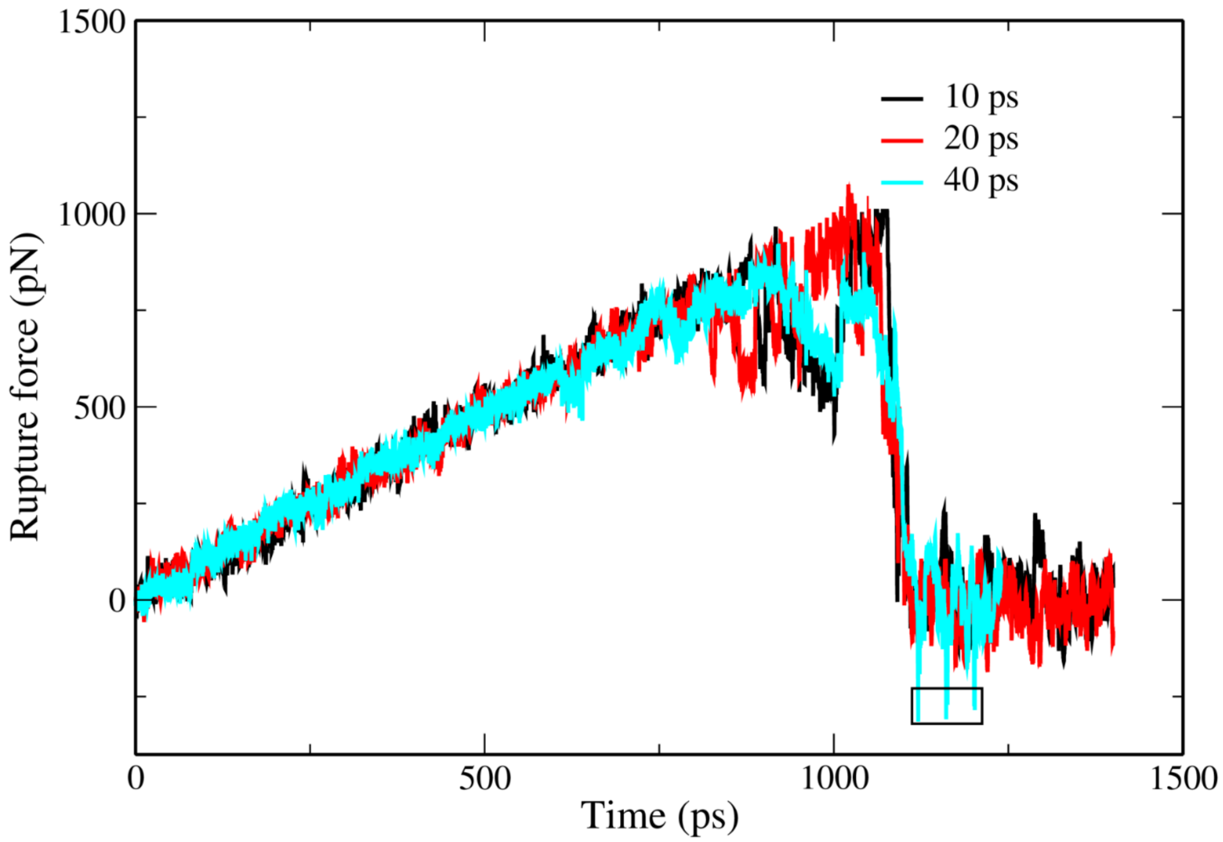

Figure S8: Force-time profiles obtained by SMD for the HupA-TcAchE complex for $\tau = 10$, 20 and 40 ps . The black box refers to the region with large variations for $\tau = 40$ ps .

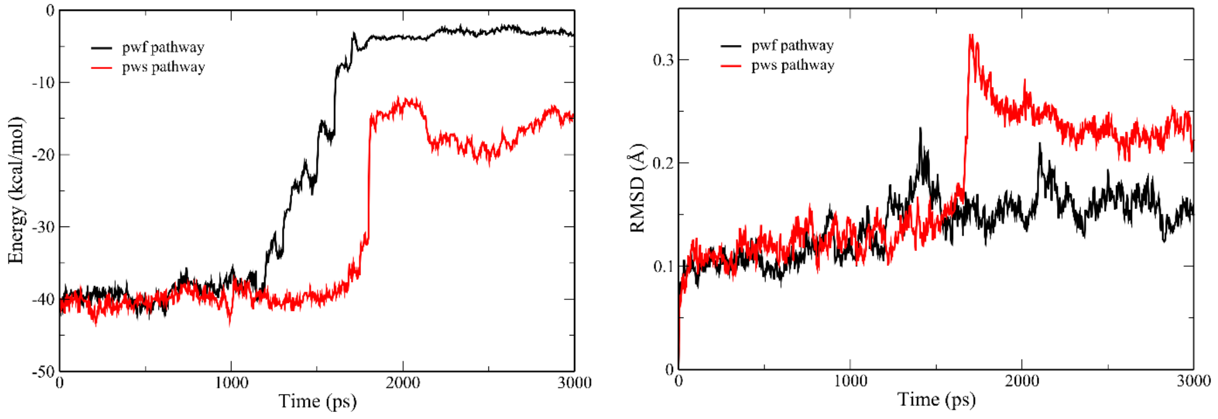

Figure S9: (Left) Non-bonded interaction energies of HupA and TcAchE along pathways pwf and pws. (Right) Time dependence of RMSD of the Ω-loop. RMSD was calculated using coordinates of back bone atoms.

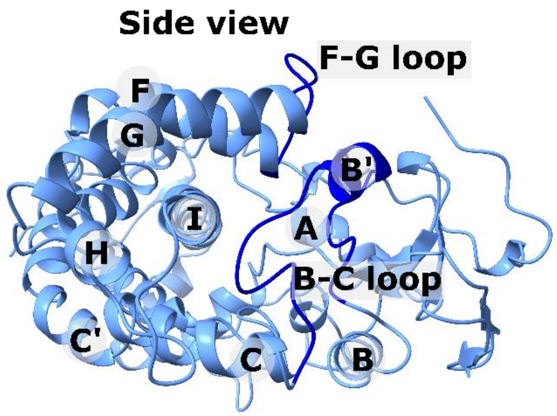

Figure S10: Structure of Cytochrome P450cam. We mark only the helixes and beta strands, which are close to the ligand dissociation pathways. The heme group is not shown.

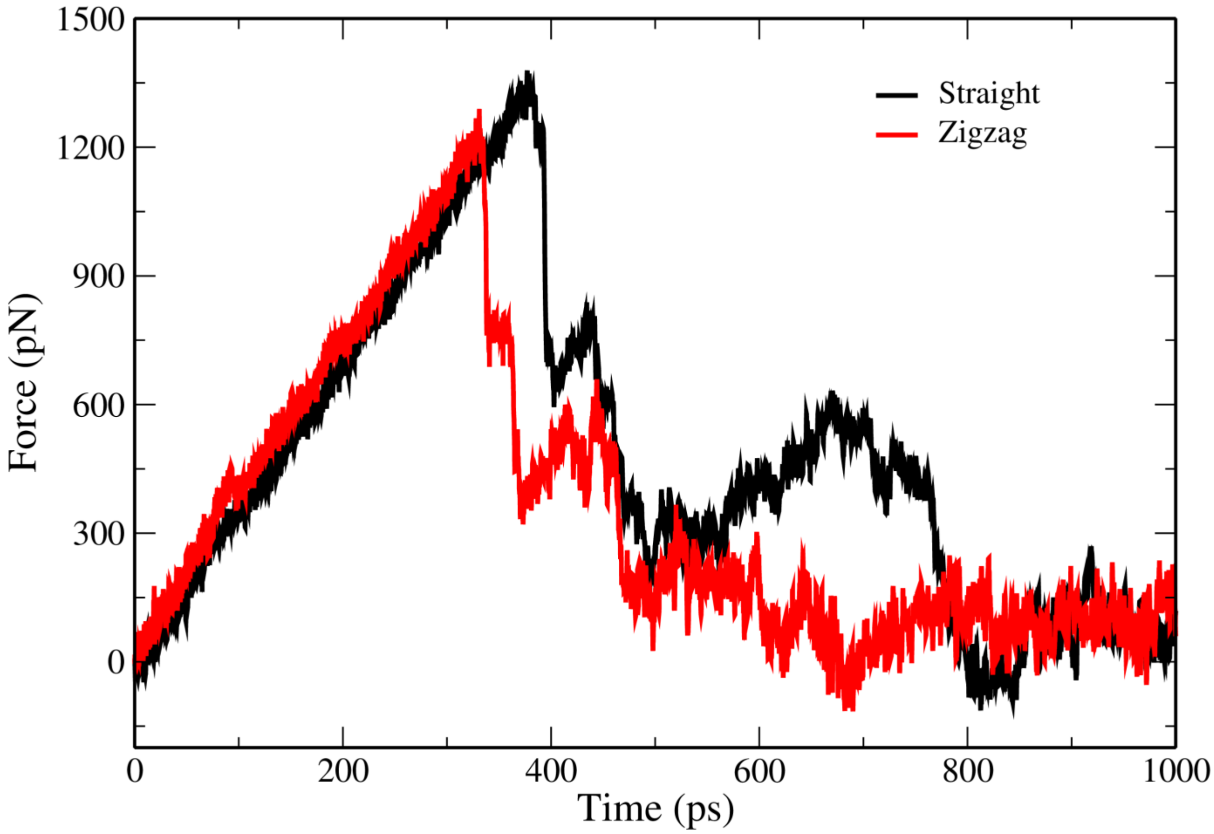

Figure S11: Force-time profiles obtained with straight and zigzag pulling for LSD1 and for ligand Aminothiazole 16 (ligand 8 in Table S1).

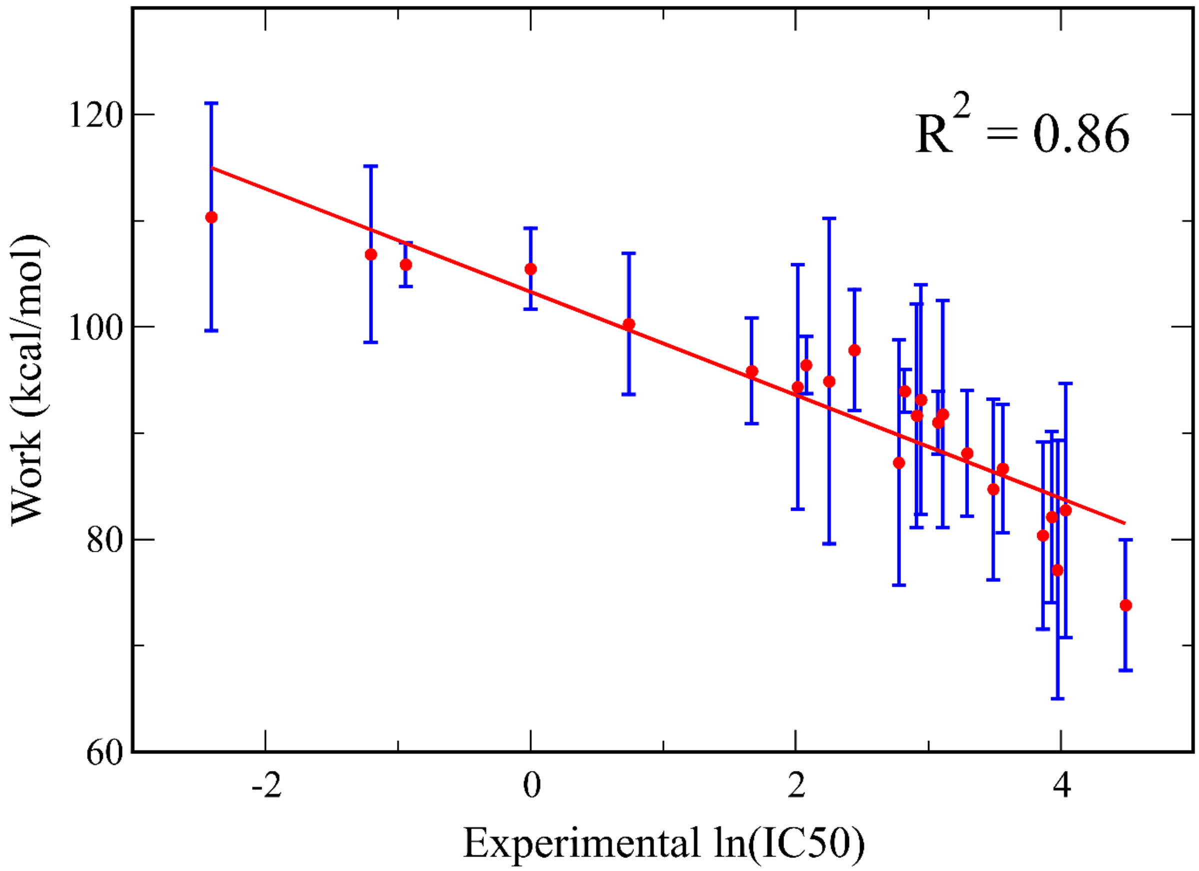

Figure S12: Pulling work as a function of the experimental ln(IC50) with IC50 measured in M. Results were obtained using SMD simulations, in which the DE-based protocol was employed to identify the zigzag pulling direction. The red line represents the data fit function. R^2 is the correlation coefficient. The results were obtained at v = 1 nm/ns and averaged over 20 SMD trajectories and error bars represent standard deviations.

References

1. Huang, Y.; Greene, E.; Stewart, T. M.; Goodwin, A. C.; Baylin, S. B.; Woster, P. M.; Casero, R. A., Inhibition of lysine-specific demethylase 1 by polyamine analogues results in reexpression of aberrantly silenced genes. Proceedings of the National Academy of Sciences 2007, 104, 8023-8028.

2. Wang, J.; Lu, F.; Ren, Q.; Sun, H.; Xu, Z.; Lan, R.; Liu, Y.; Ward, D.; Quan, J.; Ye, T., Novel histone demethylase LSD1 inhibitors selectively target cancer cells with pluripotent stem cell properties. Cancer research 2011, 71, 7238-7249.

3. Willmann, D.; Lim, S.; Wetzel, S.; Metzger, E.; Jandausch, A.; Wilk, W.; Jung, M.; Forne, I.; Imhof, A.; Janzer, A., Impairment of prostate cancer cell growth by a selective and reversible lysine-specific demethylase 1 inhibitor. International journal of cancer 2012, 131, 2704-2709.

4. Hazeldine, S.; Pachaiyappan, B.; Steinbergs, N.; Nowotarski, S.; Hanson, A. S.; Casero Jr, R. A.; Woster, P. M., Low molecular weight amidoximes that act as potent inhibitors of lysine-specific demethylase 1. Journal of medicinal chemistry 2012, 55, 7378-7391.

5. Dulla, B.; Kirla, K. T.; Rathore, V.; Deora, G. S.; Kavela, S.; Maddika, S.; Chatti, K.; Reiser, O.; Iqbal, J.; Pal, M., Synthesis and evaluation of 3-amino/guanidine substituted phenyl oxazoles as a novel class of LSD1 inhibitors with anti-proliferative properties. Organic & biomolecular chemistry 2013, 11, 3103-3107.

6. Dhanak, D. Drugging the cancer epigenome. In Proceedings of the 104th Annual Meeting of the American Association for Cancer Research, 2013; AACR Philadelphia, PA: 2013; pp 6-10.

7. Hitchin, J. R.; Blagg, J.; Burke, R.; Burns, S.; Cockerill, M. J.; Fairweather, E. E.; Hutton, C.; Jordan, A. M.; McAndrew, C.; Mirza, A., Development and evaluation of selective, reversible LSD1 inhibitors derived from fragments. MedChemComm 2013, 4, 1513-1522.

8. Sorna, V.; Theisen, E. R.; Stephens, B.; Warner, S. L.; Bearss, D. J.; Vankayalapati, H.; Sharma, S., High-throughput virtual screening identifies novel N′-(1-phenylethylidene)-benzohydrazides as potent, specific, and reversible LSD1 inhibitors. Journal of medicinal chemistry 2013, 56, 9496-9508.

9. Fiskus, W.; Sharma, S.; Shah, B.; Portier, B. P.; Devaraj, S. G.; Liu, K.; Iyer, S. P.; Bearss, D.; Bhalla, K. N., Highly effective combination of LSD1 (KDM1A) antagonist and pan-histone deacetylase inhibitor against human AML cells. Leukemia 2014, 28, 2155-2164.

10. Nobel, C. S. I.; Kimland, M.; Lind, B.; Orrenius, S.; Slater, A. F., Dithiocarbamates induce apoptosis in thymocytes by raising the intracellular level of redox-active copper. Journal of Biological Chemistry 1995, 270, 26202-26208.

11. Ye, X.-W.; Zheng, Y.-C.; Duan, Y.-C.; Wang, M.-M.; Yu, B.; Ren, J.-L.; Ma, J.-L.; Zhang, E.; Liu, H.-M., Synthesis and biological evaluation of coumarin–1, 2, 3-triazole–dithiocarbamate hybrids as potent LSD1 inhibitors. MedChemComm 2014, 5, 650-654.
